# A Hybrid Multi-Strategy Chinese Pangolin Optimization Algorithm and Its Applications

**DOI:** 10.3390/biomimetics11070480

**Published:** 2026-07-09

**Authors:** Chaochuan Jia, Yaqi Yang, Yujie Cheng, Maosheng Fu, Bao Zhou, Jiahui Liu, Yu Liu

**Affiliations:** 1 School of Electronic Information and Artificial Intelligence, West Anhui University, Lu’an 237012, China; 2 Anhui Province Intelligent Hydraulic Machinery Joint Construction Subject Key Laboratory, Lu’an 237012, China

**Keywords:** Chinese Pangolin Optimizer, multi-strategy integration, engineering applications, BP network optimization

## Abstract

To tackle the drawbacks inherent in the Chinese Pangolin Optimization (CPO) algorithm, such as uneven population initialization distribution and a tendency to fall into local optimal solutions, this paper proposes an ACDCPO algorithm that integrates boundary-adaptive contraction initialization, Cauchy inverse cumulative distribution mutation, and dynamic opposition-based learning strategies, which effectively enhances the uniformity of population distribution, improves the ability to jump out of local optimum, and strengthens the adaptive coordination between exploration and exploitation. To validate its performance, the proposed ACDCPO is compared with nine representative algorithms using the CEC2017 and CEC2022 test functions. The results verify that ACDCPO achieves remarkably higher convergence precision and stability than the comparative algorithms. In four typical engineering optimization tasks, ACDCPO shows strong constraint handling ability and engineering adaptability. In addition, based on near-infrared spectrum data, the ACDCPO algorithm optimized the BP network model for the moisture content prediction of *Dendrobium huoshanense*, and the coefficient of determination (R^2^) reached 91.211%, which verified the effectiveness of the method in practical applications.

## 1. Introduction

As modern engineering applications continue to expand, optimization problems have become increasingly complex, commonly exhibiting high dimensionality, strong nonlinearity, multiple constraints, and numerous local optima [1,2,3]. Traditional mathematical optimization methods usually rely on gradient information and often suffer from low computational efficiency and a high tendency to become trapped in local optima when dealing with complex optimization tasks [4]. In contrast, metaheuristic optimization algorithms, inspired by biological behaviors, physical laws, and natural phenomena, have become effective tools for solving complex optimization problems because they do not require gradient information and generally exhibit strong global search capability and robustness [5]. Classical algorithms, such as GA [6], PSO [7], ABC [8], BA [9], and GWO [10], have laid a solid foundation for the development of this field. In recent years, newly proposed algorithms, including PO [11], HOA [12], BKA [13], and ALA [14], have continuously emerged, further enriching the algorithmic framework and promoting the development of optimization techniques.

Although metaheuristic algorithms have been widely applied to various complex optimization problems, they still suffer from several inherent limitations. According to the No Free Lunch theorem, no optimizer can achieve the best performance across all types of problems, indicating that algorithm performance is highly dependent on the problem being solved [15]. For high-dimensional, multimodal, nonlinear, and constrained engineering optimization problems, a single search mechanism often struggles to maintain stable and superior optimization performance. Meanwhile, optimization methods have also been extended to unsupervised learning, federated learning, and multi-source data modeling, demonstrating their application potential in broader intelligent computing scenarios [16,17]. Whether in traditional engineering optimization tasks or distributed unsupervised learning scenarios, optimization algorithms commonly face challenges such as poor initial population quality, insufficient search stability, and weak global optimization capability. Most population-based optimization algorithms adopt random initialization, which can easily lead to uneven population distribution and incomplete coverage of the search space, especially in high-dimensional problems [18]. The imbalance between exploration and exploitation is another major challenge. Excessive emphasis on global exploration may reduce convergence speed, whereas excessive emphasis on local exploitation may cause premature convergence and make the algorithm stagnate in local optima [19]. In addition, fixed parameters or parameters with weak adaptive capability are difficult to match different search stages, which may reduce convergence accuracy and robustness [20].

To overcome the above limitations, many researchers have focused on integrating the advantages of different strategies [21] to balance global search and local refinement, thereby improving the comprehensive performance of algorithms [22]. For example, Cao et al., tackling the limitations of the ZOA regarding its local optimum avoidance capability and convergence speed, introduced Kent chaotic mapping to initialize the population, which optimized the spatial population distribution while enhancing the global search capability of the algorithm. They further incorporated a golden sine update mechanism to optimize search efficiency and accuracy in the later stages [23]. Wei et al. employed GNS theory to achieve uniform population initialization for the WOA and introduced a Cauchy inverse cumulative distribution strategy [24], which significantly strengthened the adaptive balance between global exploration and local exploitation [25]. In addition, X. Wang and W. Zhang embedded dynamic opposition-based learning into the Teaching-Learning-Based Optimization algorithm, effectively improving population diversity and search performance. These studies demonstrate that multi-strategy integration can significantly enhance optimization performance [26].

The CPO is a newly proposed population-based optimization algorithm inspired by the hunting behavior of Chinese pangolins in nature [27]. It simulates the process by which pangolins perceive the odor concentration of ants and flexibly switch between different foraging behaviors, providing a new search framework for solving optimization problems. Although CPO has shown promising optimization performance, it still has several limitations when solving complex problems [28]. First, the random initialization mechanism may result in uneven population distribution and slow convergence. Second, the algorithm lacks an effective mechanism to adaptively balance global exploration and local exploitation. Third, population diversity decreases rapidly in the later iterations, making the algorithm prone to premature convergence and local optima [29]. The ablation experimental results further verify the limitations of the original CPO algorithm. Specifically, the original CPO still has considerable room for improvement in terms of convergence accuracy, population diversity maintenance, and the ability to escape local optima. Meanwhile, the results also demonstrate that the three proposed improvement strategies can synergistically enhance the robustness and convergence performance of the algorithm.

To address these limitations, this paper proposes a multi-strategy improved ACDCPO algorithm. The main improvements are as follows:

1. The boundary-adaptive contraction initialization strategy is introduced at the population initialization stage. Instead of initializing all individuals within the complete search interval, this strategy adaptively shrinks the initialization range according to the search scale of each dimension, thereby reducing excessive dispersion in large-range dimensions while maintaining sufficient coverage in small-range dimensions. This improves the rationality of the initial population distribution and provides more stable starting points for subsequent search.

2. The Cauchy inverse cumulative distribution mutation is embedded into the position update process. Owing to the sharp peak and heavy-tailed characteristics of the Cauchy distribution, this mechanism supports local exploitation around promising solutions while increasing the probability of large perturbations, thereby improving CPO’s ability to escape local optima without weakening local refinement.

3. Dynamic opposition-based learning is applied after population updating. Unlike conventional opposition-based learning with fixed global boundaries, the proposed strategy generates opposite solutions based on the real-time population distribution, making candidate solutions more relevant to the current search region. This helps explore uncovered areas, maintain population diversity, and reduce invalid solutions.

To verify the effectiveness of the proposed ACDCPO algorithm, comparative experiments are conducted on the CEC2017 and CEC2022 standard benchmark test suites [30], four engineering optimization cases, and a practical moisture content prediction task for *Dendrobium huoshanense* based on a BP neural network. The experimental results show that ACDCPO effectively overcomes the limitations of the original CPO in complex optimization problems through multi-strategy fusion. It exhibits superior performance in high-dimensional, multimodal, and constrained engineering optimization problems, verifying the effectiveness of the proposed improvement strategies and providing a new technical approach for the practical engineering application of bio-inspired optimization algorithms.

The remainder of this paper is organized as follows. Section 2 presents the complete procedure of the proposed ACDCPO algorithm and its corresponding improvement strategies. Section 3 conducts numerical simulation experiments and comparative analysis based on the CEC2017 and CEC2022 benchmark test suites. Section 4 verifies the effectiveness of ACDCPO using four practical engineering optimization cases and the BP-network-based moisture content prediction task for *Dendrobium huoshanense*. Section 5 summarizes the conclusions and discusses future research directions.

## 2. The Multi-Strategy Chinese Pangolin Optimizer

The ACDCPO algorithm combines three strategies: initialization based on adaptive contraction of the boundary, the Cauchy inverse cumulative distribution strategy, and dynamic opposition-based learning. Together, these strategies solve the problem of uneven distribution of candidate solutions in the search space, enhance the ability of the algorithm to overcome local optima, and adaptively coordinate exploration with exploitation. The detailed process of each stage is presented below.

### 2.1. Initialization Based on Adaptive Boundary Contraction

To avoid the excessively scattered distribution of initial individuals caused by traditional random initialization in high-dimensional or multi-scale optimization problems, this paper proposes a boundary-adaptive shrinkage initialization strategy. This strategy adaptively adjusts the initialization interval according to the length of the search range of each dimensional variable. For dimensions with a large search range, a stronger shrinkage strength is assigned to reduce the retained interval ratio, so as to mitigate the excessive dispersion of initial samples on large-scale dimensions. For dimensions with a narrow search range, a relatively wide initialization interval is reserved to maintain sufficient search coverage capacity.

Let the population size be *N* and the dimension of the optimization problem be *D*. The lower and upper search bounds of the *j*-th dimension are denoted as *lb_j_* and *ub_j_*, respectively. The search interval length of the *j*-th dimension is defined as:

(1) $$L_{j}=ub_{j}-lb_{j},j=1,2,\ldots ,D$$

The maximum interval length among all dimensions is defined as:

(2) $$L_{\max}=\max_{1\leq j\leq D}L_{j}$$

The shrinkage strength of the *j*-th dimension is defined as:

(3) $$k_{j}=\alpha \frac{L_{j}}{L_{\max}}$$

where *α* denotes the boundary shrinkage control parameter. The retained ratio of the shrunk interval can thus be derived as:

(4) $$\rho_{j}=1-k_{j}=1-\alpha \frac{L_{j}}{L_{\max}}$$

It can be observed from the above formulas that a larger interval length *L_j_* of a dimension corresponds to a higher shrinkage strength *k_j_* and a larger shrinkage amplitude of this dimension. Meanwhile, a smaller retained interval ratio *ρ_j_* indicates a narrower available initialization interval. This design realizes strong shrinkage for dimensions with wide value ranges and wide reserved initialization intervals for dimensions with narrow value ranges. Given 0 < *α* < 1 and 0 < *L_j_* ≤ *L*_max_, the following relation holds: 1 − *α* ≤ *ρ_j_* <1.

The center point of the search interval for the *j*-th dimension is calculated as:

(5) $$c_{j}=\frac{ub_{j}+lb_{j}}{2}$$

The new lower and upper bounds of the *j*-th dimension after shrinkage are:

(6) $$lb_{j}^{*}=c_{j}-\frac{\rho_{j}L_{j}}{2},ub_{j}^{*}=c_{j}+\frac{\rho_{j}L_{j}}{2}$$

The initial position of the *i*-th individual on the *j*-th dimension is generated based on the shrunk interval:

(7) $$X_{i,j}=lb_{j}^{*}+r_{i,j}\left(ub_{j}^{*}-lb_{j}^{*}\right)$$

Its equivalent transformation is written as:

(8) $$X_{i,j}=c_{j}+\left(2r_{i,j}-1\right)\frac{\rho_{j}L_{j}}{2}$$

where *r_i,j_* follows the uniform distribution *U*(0,1), *i* = 1, 2, …, *N*. *j* = 1, 2, …, *D*. Combining 0< *ρ_j_* ≤ 1, the constraint relation can be deduced as: *lb_j_* ≤ $lb_{j}^{*}$ ≤ *X_i,j_* ≤ $ub_{j}^{*}$ ≤ *ub_j_*.

This formula indicates that all individuals generated by the proposed initialization strategy always lie within the shrunk interval and will never exceed the original search boundaries. According to the setting form of the upper and lower bounds of optimization variables, the initialization process is designed separately for two scenarios: unified bounds and dimension-independent bounds.

1. Scenario of Unified Upper and Lower Bounds:

When *size*(*ub*,2) = 1, all dimensions share identical upper and lower bounds, meaning variables of all dimensions take values within the unified search interval [*lb*, *ub*]. In this case, all dimensions possess consistent search scales, and differentiated shrinkage ratios across dimensions are unnecessary. A fixed interval retention ratio of *ρ* = 0.8 is therefore adopted to uniformly shrink the initialization intervals.

The interval center point is:

(9) $$c=\frac{ub+lb}{2}$$

The initial position of the *i*-th individual on the *j*-th dimension is:

(10) $$X_{i,j}=c+\left(2r_{i,j}-1\right)\frac{\rho \left(ub-lb\right)}{2}$$

where *ρ* = 0.8, *r_i,j_*~*U*(0,1). This formula means that initial individuals are generated within 80% of the original search range centered on the interval midpoint for all dimensions.

2. Scenario of Independent Upper and Lower Bounds for Each Dimension (*size* (*ub*,2) > 1):

The adaptive shrinkage strategy proposed in this paper is adopted: the interval retention ratio *ρ_j_* is dynamically solved according to the interval length *L_j_* of each dimension, and initial individuals are generated within the shrunk interval [$lb_{j}^{*},ub_{j}^{*}$]. In this paper, the boundary shrinkage control parameter is set as *α* = 0.3. This parameter setting avoids two extreme cases: excessive shrinkage will lead to insufficient search-space coverage of the initial population, while zero shrinkage will result in overly dispersed initial solutions for high-dimensional large-scale problems. It can be obtained that *ρ_j_* ∈ [0.7, 1), that is, the initialization interval of each dimension is adaptively shrunk to 70–100% of the original interval.

The initial position of the *i*-th individual on the *j*-th dimension generated from the shrunk interval is:

(11) $$X_{i,j}=\frac{ub_{j}+lb_{j}}{2}+\left(2r_{i,j}-1\right)\frac{ub_{j}-lb_{j}}{2}\left[1-0.3\frac{ub_{j}-lb_{j}}{\max_{j=1,\ldots ,D}\left(ub_{j}-lb_{j}\right)}\right]$$

This strategy reduces invalid dispersion of individuals on wide-range dimensions while guaranteeing sufficient coverage of the global search space. It improves the compactness and rationality of the initial population distribution and provides more stable starting points for subsequent iterative search.

### 2.2. Luring Behavior

In the ACDCPO algorithm, the luring behavior is modeled into two phases, namely the attraction and capture phase and the movement and foraging phase. The execution condition is $C_{M}\left(t\right)\geq 0.2$ and *r*_1_ ≤ 0.5, where *C_M_*(*t*) denotes the odor concentration in the *t*-th iteration, and *r*_1_ is a random number in the range [0, 1].

Stage 1: Attraction and Capture Stage. At this stage, ants are captured by gradually approaching the pangolin, attracted by the gas released by the pangolin. The relevant formula is as follows:

(12) $$H_{A}\left(t\right)=\left|m\times S_{A}\left(t\right)-S_{M}\left(t\right)\right|$$

(13) $$S_{A}\left(t+1\right)=S^{*}\left(t\right)+S_{A}\left(t\right)-A_{1}\times H_{A}\left(t\right)$$

*H_A_*(*t*) represents the separation between the ant and the pangolin at the *t*-th iteration. *m* denotes the gas trajectory factor, *S_A_*(*t*) denotes the position of the ant at the *t*-th iteration, and *S_M_*(*t*) denotes the position of the Chinese pangolin at the *t*-th iteration. S^*^(*t*) represents the optimal position within the population at the *t*-th iteration, and *A*_1_ is the energy fluctuation factor.

(14) $$m=\sqrt{m_{x}^{2}+m_{y}^{2}+m_{z}^{2}}$$

Equation (14): $m_{x}$, $m_{y}$, and $m_{z}$ represent the variations in scent position in the *x*, *y*, and *z* directions at each iteration, respectively.

(15) $$A_{1}=2\times E\times rand\left(\right)-E$$

(16) $$E=\exp\left(-\lambda \times V_{O2}\times t\times \left(1+Fatigue\right)\right)$$

(17) $$Fatigue=\log\left(\frac{t\times \pi +T}{T}\right)$$

$A_{1}$ represents the energy fluctuation factor, *E* denotes the energy dissipation coefficient, λ represents the energy correction factor, $V_{O2}$ denotes the oxygen uptake, and *Fatigue* signifies the fatigue index factor.

Stage 2: Movement and Feeding Stage. At this stage, the ants have been captured by the pangolin and move towards the best position. The relevant formulas are given as follows:

(18) $$H_{M}\left(t\right)=\left|C_{1}\times H_{A}\left(t\right)-S_{M}\left(t\right)\right|+L^{*}\times \left(1-\frac{t}{T}\right)$$

(19) $$S_{M}\left(t+1\right)=S^{*}\left(t\right)+S_{M}\left(t\right)-A_{1}\times H_{M}\left(t\right)$$

(20) $$C_{1}=2-t\times \left(\frac{2}{T}\right)$$

Equation (18) primarily simulates the relative positional changes between the Chinese pangolin and the closest river. Here, *HM*(*t*) represents the separation distance between the pangolin and the nearest river in the *t*-th iteration. *C*_1_ denotes the rapid decay factor, calculated via Equation (20). L* represents the Lévy flight function used to simulate the pangolin’s stochastic movement. Equation (19) primarily simulates the positional changes as the Chinese pangolin guides the ant colony toward the river under the influence of energy fluctuations.

The Chinese pangolin will update its position through Formula (21) in the whole stage of luring behavior, $r_{2}$ is a random number uniformly distributed in the interval [0, 1]:

(21) S∗t+1=SMt+SAt2+r1×r2×rand×sinSAt×etT4π×tanSMt×e4π2T

### 2.3. Predatory Behavior

Under the conditions of *C_M_* ≤ 0.7|| *r*_1_ > 0.5, the stage of predation behavior is divided into three stages according to the value range of *C_M_*, and the specific information is as follows.

Stage 1: Search and Localization Stage ($0\leq C_{M}<0.3$). During this process, the pangolin’s random walk follows a Lévy flight function to search for and locate the ant nest. The relevant formulas are given as follows:

(22) $$H_{M}\left(t\right)=\left|a\times S_{M}\left(t\right)-S^{*}\left(t\right)\right|$$

(23) $$S_{M}\left(t+1\right)=S^{*}\left(t\right)-A_{1}\times \left|S_{M}\left(t\right)-\exp\left(-a\right)\times \sin\left(rand\times \pi \right)\times H_{M}\left(t\right)\right|$$

Equation (22) represents the change in the distance between the position of the Chinese pangolin after a random walk and the position of the ant nest in the *t*-th iteration. Equation (23) represents the change in position during the rapid approach to the ant nest, influenced by energy consumption.

Stage 2: Rapid Approach Stage ($0.3\leq C_{M}<0.6$). During this stage, the Chinese pangolin rapidly approaches the location of the ant nest along the scent trajectory. The relevant formulas are given as follows:

(24) $$H_{M}\left(t\right)=\left|a\times S_{M}\left(t\right)-S^{*}\left(t\right)\right|$$

(25) $$S_{M}\left(t+1\right)=S^{*}\left(t\right)-A_{1}\times \left|S_{M}\left(t\right)-\exp\left(-a\right)\times \sin\left(rand\times \pi \right)\times H_{M}\left(t\right)\right|$$

Equation (24) describes the distance between the pangolin and the ant nest as the pangolin moves along the odor trajectory. Equation (25) represents the process of rapidly approaching the ant nest affected by energy consumption, odor trajectory, and random walk behavior.

Stage 3: Digging and Feeding Stage ($C_{M}\geq 0.6$). At this phase, the pangolin has determined the position of the ant nest. The relevant formulas are given as follows:

(26) $$H_{M}\left(t\right)=\left|C_{1}\times S_{M}\left(t\right)-S\left(t\right)\right|$$

(27) $$S_{M}\left(t+1\right)=S^{*}\left(t\right)+A_{1}\times \left|S_{M}\left(t\right)-H_{M}\left(t\right)\right|$$

During the complete predation process, Equation (28) is applied to update the optimal position of the Chinese pangolin.

(28) $$S^{*}\left(t+1\right)=C_{1}\times S_{M}\left(t\right)$$

### 2.4. Cauchy Inverse Cumulative Distribution Mutation

The Cauchy distribution features a sharp peak and heavy-tailed properties. The sharp peak facilitates local exploitation around the current high-quality solutions, while the heavy tail can generate large disturbances with a high probability, enabling the algorithm to escape local optima [31]. To strengthen the global search capability of CPO, this paper introduces a mutation mechanism based on the inverse cumulative distribution of Cauchy distribution during the position update process.

The probability density function of the Cauchy distribution is expressed as:

(29) $$Y\left(x;b,c\right)=\frac{1}{\pi c\left[1+{\left(\frac{x-b}{c}\right)}^{2}\right]}$$

The cumulative distribution function is:

(30) $$\hat{Y}\left(x;b,c\right)=\frac{1}{\pi }\arctan\left(\frac{x-b}{c}\right)+\frac{1}{2}$$

The inverse cumulative distribution function is:

(31) $$Y{\left(x;b,c\right)}^{-1}=b+c\tan\left(\pi \times \left(p-\frac{1}{2}\right)\right)$$

where *p*~*U*(0,1). When *b* = 0 and *c* = 1, the Cauchy disturbance term is written as:

(32) $$C_{i,j}=\tan\left[\pi \left(p_{i,j}-\frac{1}{2}\right)\right]$$

The mutation update formula is defined as:

(33) $$S^{*}\left(t+1\right)=\left\{\begin{array}{c} \begin{array}{cc} S^{*}\left(t\right), & r\leq 0.5 \end{array}\\ \begin{array}{cc} S^{*}\left(t\right)\times \tan\left[\pi \left(p_{i,j}-\frac{1}{2}\right)\right], & r>0.5 \end{array} \end{array}\right.$$

Here, *r*~*U*(0,1) is only used to determine whether to perform Cauchy mutation, and *p_i,j_*~*U*(0,1) is adopted to generate Cauchy-distributed disturbances.

### 2.5. Dynamic Opposition-Based Learning

To address the insufficient diversity of the initial population and the tendency to become trapped in local optima, this study introduces a dynamic opposition-based learning strategy. Unlike conventional opposition-based learning methods that rely on fixed global boundaries, the proposed strategy dynamically determines the opposition search range according to the real-time distribution of the population. Specifically, opposite solutions are generated based on the current population boundaries, and high-quality individuals are retained through fitness-based selection while the global best solution is updated accordingly.

(34) $$S^{*}\left(t+1\right)=S^{*}\left(t\right)+w\times rand\times \left(rand\times S_{j}^{O}\left(t\right)-S^{*}\left(t\right)\right)$$

*rand* is a random variable ranging from 0 to 1, and *w* represents a weighting factor greater than 0. *S^O^* is obtained by opposition-based learning, and $S^{*}\left(t+1\right)$ denotes the best population position generated through dynamic opposition-based learning.

(35) $$S_{j}^{O}\left(t\right)=ub_{j}+lb_{j}-S_{j}^{*}\left(t\right),j=1,2,\ldots ,D$$

The value of the optimal position of the population in dimension *D* is between *lb_j_* and *ub_j_*, and the specific search space is as follows:

(36) $$\begin{array}{c} lb_{j}=\min S_{j}^{*}\left(t\right)\\ ub_{j}=\max S_{j}^{*}\left(t\right) \end{array}$$

This strategy replaces the fixed global-boundary generation approach with dynamic boundary opposition-based learning. Based on the real-time boundaries of the population, new solutions are generated at symmetric positions to effectively supplement uncovered regions, thereby avoiding invalid solutions and improving search efficiency. Essentially, it functions as a symmetric solution supplementation mechanism that enhances the algorithm’s capability to escape local optima and strengthens global optimization ability.

### 2.6. Computational Complexity Analysis

To evaluate the additional computational cost introduced by the proposed enhancement mechanisms, this section analyzes the computational complexity of the original CPO and the proposed ACDCPO. Let *N* denote the population size, *T* the maximum number of iterations, *D* the problem dimension, and *C_f_* the computational cost of one fitness evaluation.

For the original CPO, the initialization stage generates an *N* × *D* population matrix, resulting in a complexity of *O*(*ND*). In each iteration, boundary checking requires *O*(*ND*), fitness evaluation requires *O*(*NC_f_*), and position updating mainly involves vector operations in a *D*-dimensional search space, requiring *O*(*ND*). Therefore, the total computational complexity of CPO can be expressed as *O*(*ND*) + *O*(*T*(*NC_f_* + *ND*)); ignoring lower-order terms, the overall complexity of CPO is *O*(*TN*(*C_f_* + *D*)).

For ACDCPO, the boundary-adaptive contraction initialization also requires *O*(*ND*), which is the same order as the standard initialization. During each iteration, ACDCPO retains the original boundary checking, fitness evaluation, and position update procedures of CPO. In addition, the Cauchy inverse cumulative distribution mutation generates *D*-dimensional perturbations for the population and performs boundary handling, resulting in a computational cost of *O*(*ND*). The dynamic opposition-based learning mechanism calculates the dynamic boundaries of the current population, generates the opposite population, and performs additional fitness evaluations. According to the current implementation, this mechanism introduces approximately 2*N* extra fitness evaluations in each iteration, and its complexity is *O*(*ND* + 2*NC_f_*).

Thus, the per-iteration complexity of ACDCPO can be written as *O*(*NC_f_* + *ND*) + *O*(*ND*) + *O*(*ND*) + *O*(*ND* + 2*NC_f_*), which can be simplified as *O*(3*NC_f_* + 4*ND*). After *T* iterations, the total complexity of ACDCPO is *O*(*T*(3*NC_f_* + 4*ND*)). By ignoring constant coefficients, its asymptotic complexity can also be expressed as *O*(*TN*(*C_f_* + *D*)).

Therefore, ACDCPO has the same asymptotic complexity order as the original CPO. When simple benchmark functions are used and *C_f_* = *O*(*D*), the complexity of both CPO and ACDCPO can be further simplified to *O*(*TND*). However, in terms of actual computational cost, ACDCPO requires more fitness evaluations than CPO. Specifically, CPO performs approximately *N* fitness evaluations per iteration, whereas ACDCPO performs approximately 3*N* fitness evaluations due to the additional dynamic opposition-based learning strategy. Hence, the main extra computational cost of ACDCPO comes from the dynamic opposition-based learning mechanism, while the boundary-adaptive initialization and Cauchy mutation do not change the asymptotic complexity order. This moderate increase in computational cost is acceptable because these mechanisms improve population diversity, enhance global exploration, and reduce the risk of premature convergence.

**Figure 1:**
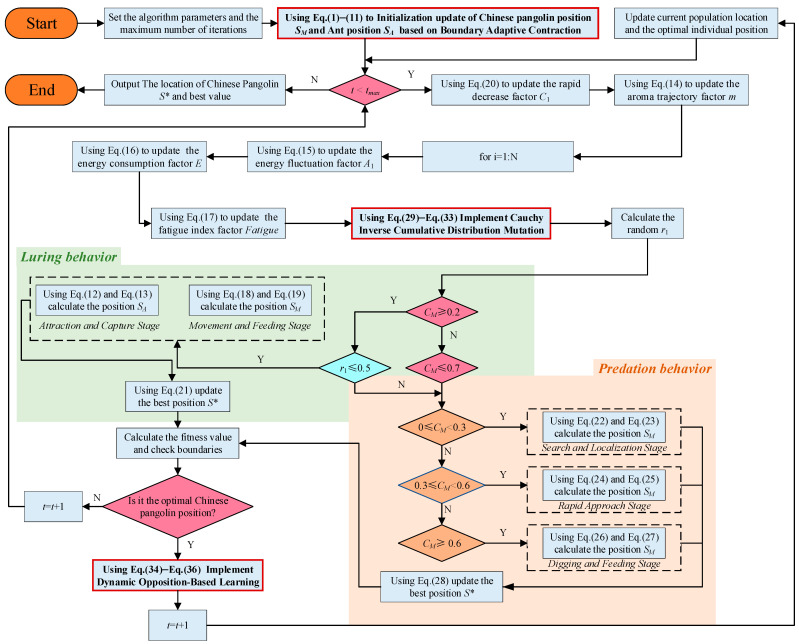
The flowchart of the ACDCPO.

To clearly illustrate the implementation logic and key steps of the ACDCPO algorithm, this paper presents the algorithm’s pseudocode (as presented in Algorithm 1) and the associated flowchart (as presented in Figure 1). The ACDCPO algorithm first initializes the population using a boundary-adaptive contraction strategy and then evaluates the global optimal position of the initial population. Afterward, the algorithm enters the primary loop, with *Max*_*iter* serving as the termination condition. In each iteration, relevant parameters are pre-updated first, and the algorithm enhances the global search capability by performing the Cauchy inverse cumulative distribution mutation operation. If *C_M_* ≥ 0.2 and *r*_1_ ≤ 0.5, the luring behavior is triggered; otherwise, the predation behavior is executed. Finally, population diversity is optimized via dynamic opposition-based learning, and the next iteration begins after the iteration counter is updated. The algorithm terminates when the maximum iteration number *Max_iter* is reached, and then it outputs the optimal pangolin position S* and its corresponding fitness value.
**Algorithm 1: ACDCPO****Outputs:** The location of the Chinese pangolin $S^{*}$ and Best Fitness
1: **Initialization Based on Boundary-Adaptive Contraction, and evaluate the initial population using Equations (1)–(11)**
2: **while (*t* < *Max*_*iter*) do**3:   **for i = SearchAgents_no do**4:     The parameter factor used for updating behavior transition5:     **Using Equations (29)–(33), update the position of the Chinese pangolins using Cauchy inverse cumulative distribution mutation**6:      **if** ($C_{M}\geq 0.2\&\&r_{1}>0.5$) **then**7:        Update the location using Equations (12) and (13)//***Capture Stage***8:        Update the location using Equations (18) and (19)//***Feeding Stage***9:        Using Equation (21) update the best position $S^{*}$
10:      **else if** ($C_{M}\leq 0.7||r_{1}>0.5$) **then**11:        **if** ($0\leq C_{M}<0.3$) **then //*Search and Localization Stage***12:        Update the location using Equations (22) and (23)13:        **else if** ($0.3\leq C_{M}<0.6$) **then //*Rapid Approach Stage***14:        Update the location using Equations (24) and (25)15:        **else if** ($C_{M}\geq 0.6$) **then //*Digging and Feeding Stage***16:        Update the location using Equations (26) and (27)17:        Using Equation (28), update the best position S^*^18:        **end if**19:      **end if**20:   **end for**21:   Calculate the fitness values of the Chinese pangolin and apply boundary checking22:  **Using Equations (34)–(36), update the position of the Chinese pangolin using dynamic reverse learning**23:   *t* = *t* +124: **end while**25: **return** Best Position $S^{*}$ and Best Fitness

## 3. Numerical Experiments and Comparative Analysis

This paper employs the CEC2017 and CEC2022 test function sets as evaluation benchmarks. These datasets encompass various function types, which can comprehensively evaluate the performance of the ACDCPO algorithm. To enhance the comparability and reference value of the evaluation, other optimization algorithms are introduced as benchmarks. These include the GA, WOA [32], CFOA [33], COA [34], AOA [35], LCA [36], MShOA [37], and SFOA [38]. For the purpose of reducing the influence of randomness on experimental results and improving result reliability, all experiments were repeated 30 times independently, and the average value of the performance index was used as the final evaluation result.

Regarding the experimental environment, this paper employs an AMD Ryzen 7 4800 H processor paired with an NVIDIA GeForce RTX 1650Ti graphics card, running on the Windows 10 64-bit operating system. All algorithms are implemented on the MATLAB 2024a platform. The maximum iterations and population size are set to 1000 and 30, respectively. The CEC2017 test function was configured with 50 dimensions, while the CEC2022 test function used 20 dimensions to ensure comprehensive and objective evaluation. Except for the proposed enhancement mechanisms in ACDCPO, the algorithm-specific parameters of all comparison algorithms were set according to the values recommended in their original references or source codes. No additional manual parameter tuning was performed for any comparison algorithm.

### 3.1. Ablation Studies of the ACDCPO Algorithm

To evaluate the effect of each newly introduced mechanism on model performance, ablation experiments were performed using MATLAB (2024a). The original CPO algorithm was compared with three improved variants: ACPO, CCPO, and DCPO. ACPO employs boundary-adaptive contraction initialization, CCPO employs Cauchy inverse cumulative distribution mutation, and DCPO utilizes dynamic reverse learning. Moreover, ACCPO combines boundary-adaptive contraction initialization and Cauchy inverse cumulative distribution mutation, ADCPO integrates boundary-adaptive contraction initialization with dynamic reverse learning, and CDCPO incorporates Cauchy inverse cumulative distribution mutation and dynamic reverse learning. The population size was uniformly set to 30, and the maximum number of iterations was set to 300. The ranking of each algorithm was calculated based on the average fitness values obtained from 30 independent runs on different test functions. Each group of test functions was independently repeated 30 times to reduce experimental errors caused by randomness and improve the reliability of the statistical results.

**Figure 2:**
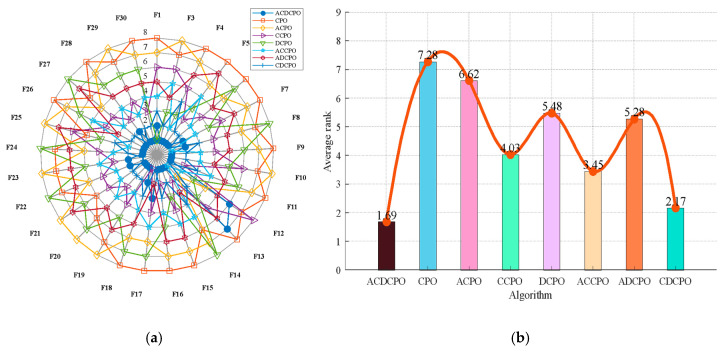
Results of the ablation study based on CEC2017. (**a**) Radar chart. (**b**) Diagram of average ranking.

Figure 2 presents the ablation results of ACDCPO. As shown in Figure 2a, the complete ACDCPO algorithm achieves the most stable ranking distribution among all variants, ranking first on 19 functions and second on seven functions, which indicates its strongest overall robustness. Figure 2b further shows that ACDCPO obtains the best average ranking of 1.69 among 29 benchmark functions, followed by CDCPO, ACCPO, CCPO, ADCPO, DCPO, ACPO, and the original CPO.

The ablation results demonstrate that the three improvement strategies contribute differently to the overall performance. Compared with the original CPO, ACPO improves the average ranking from 7.28 to 6.62, indicating that the adaptive boundary contraction initialization can improve the initial population quality, although its independent effect is limited. CCPO achieves an average ranking of 4.03, showing that Cauchy inverse cumulative distribution mutation provides the most significant single-strategy contribution by enhancing the ability to escape local optima. DCPO obtains an average ranking of 5.48, suggesting that dynamic opposition-based learning improves population diversity but is less effective than Cauchy mutation when used alone.

The results of dual-strategy variants further confirm the complementarity among the three mechanisms. ACCPO, ADCPO, and CDCPO achieve average rankings of 3.45, 5.28, and 2.17, respectively, among which CDCPO performs best. This indicates that Cauchy mutation and dynamic opposition-based learning have strong synergistic effects. Overall, the complete ACDCPO achieves the best performance because the three strategies jointly improve initial population quality, local optimum escaping ability, and population diversity maintenance.

### 3.2. Performance Evaluation and Analysis on CEC2017 Benchmark Functions

The CEC2017 dataset consists of 29 test functions: unimodal functions (*F*_1_ and *F*_3_), which have only one global optimum and are used to evaluate the local exploitation ability and convergence speed of algorithms; simple multimodal functions (*F*_4_–*F*_10_), which contain multiple local optima and are used to assess the capability of global search and escape from local optima; hybrid functions (*F*_11_–*F*_20_), which are constructed from a weighted combination of 3–6 unimodal and multimodal functions in different subspaces, forming diverse search regions; and composition functions (*F*_21_–*F*_30_), which are built through nonlinear combinations of multiple hybrid or basic functions and represent the most challenging type of functions in the CEC2017 benchmark suite. Table 1 presents the classification information of the CEC2017 test suite functions.

#### 3.2.1. Core Parameter Analysis on CEC2017 Benchmark Functions

Table 2 compares the performance of all algorithms on the CEC2017 benchmark functions (*F*_1_–*F*_30_) using four indicators: minimum value (min), standard deviation (std), and average value (avg). Overall, ACDCPO ranks first on 27 out of 29 functions and second only on *F*_15_ and *F*_19_, demonstrating the best comprehensive performance among all compared algorithms.

For the unimodal functions *F*_1_ and *F*_3_, ACDCPO achieves the best minimum values, indicating strong exploitation ability and convergence accuracy. Taking *F*_1_ as an example, ACDCPO obtains a minimum value of 5.90 × 10^6^, outperforming the second-ranked CPO (1.20 × 10^7^). Compared with AOA and GA, its optimization accuracy improves by four to five orders of magnitude, suggesting that the adaptive boundary contraction initialization effectively improves the initial population quality and helps the algorithm approach the optimal region more efficiently.

For the simple multimodal functions *F*_4_–*F*_10_, ACDCPO ranks first on all functions. On *F*_9_ and *F*_10_, it achieves the best average values while maintaining relatively low standard deviations, indicating a good balance between exploration and exploitation. In contrast, algorithms such as GA and SFOA are more prone to premature convergence, resulting in lower accuracy and weaker stability on multimodal landscapes.

For the hybrid functions *F*_11_–*F*_20_, ACDCPO obtains the best performance on most functions, while the original CPO ranks first only on *F*_15_ and *F*_19_. This result is consistent with the No Free Lunch theorem, which indicates that no optimizer can dominate all problem landscapes. Nevertheless, ACDCPO still shows stronger overall competitiveness on hybrid functions. For example, on *F*_13_, although CPO obtains a smaller minimum value, ACDCPO achieves better average values, indicating a more stable search process and lower sensitivity to random fluctuations.

For the composition functions, *F*_21_–*F*_30_, ACDCPO ranks first on all functions. Since these functions involve shifting, rotation, weighting, and nonlinear combination, they impose higher requirements on robustness and population diversity maintenance. On *F*_25_, ACDCPO achieves the best minimum and average values, with 3.08 × 10^3^ and 3.19 × 10^3^, respectively, and ranks first among all compared algorithms. Although its standard deviation is slightly higher than that of CPO, it remains at a low level and is much smaller than that of most other algorithms, such as SFOA (1.70 × 10^4^). These results indicate that ACDCPO maintains high optimization accuracy and stable search performance on complex composition functions, further demonstrating the effectiveness of dynamic opposition-based learning in preserving population diversity and enhancing robustness.

#### 3.2.2. Convergence Analysis on CEC2017 Benchmark Functions

**Figure 3:**
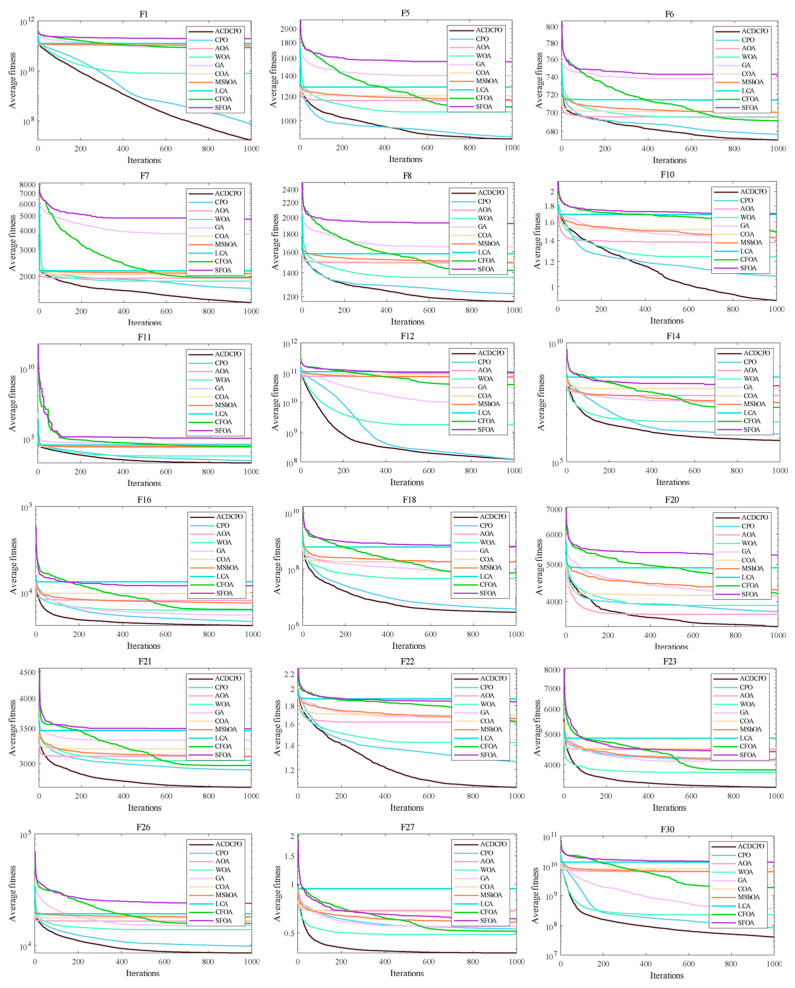
Convergence curves of selected test functions in CEC2017.

Analysis of the convergence curves in Figure 3 shows that the proposed ACDCPO algorithm achieves superior convergence performance on most benchmark functions. For unimodal and simple multimodal functions (*F*_1_, *F*_5_, and *F*_6_), ACDCPO rapidly reduces fitness values in early iterations and maintains steady convergence in the later stage, with fast convergence speed and high accuracy.

For complex hybrid and composition functions (*F*_12_, *F*_14_, and *F*_30_) with numerous local optima, comparison algorithms such as AOA and COA easily stagnate at high fitness levels and their curves flatten prematurely. In contrast, ACDCPO keeps decreasing fitness values throughout iterations and finally converges to a much lower level, proving its stronger ability to escape local optima and perform global optimization.

Overall, the convergence results verify the effectiveness of the multi-strategy improvement mechanism. With the synergy of three strategies, ACDCPO achieves faster convergence, higher optimization accuracy, and more stable search performance across different types of test functions.

#### 3.2.3. Stability Analysis on CEC2017 Benchmark Functions

**Figure 4:**
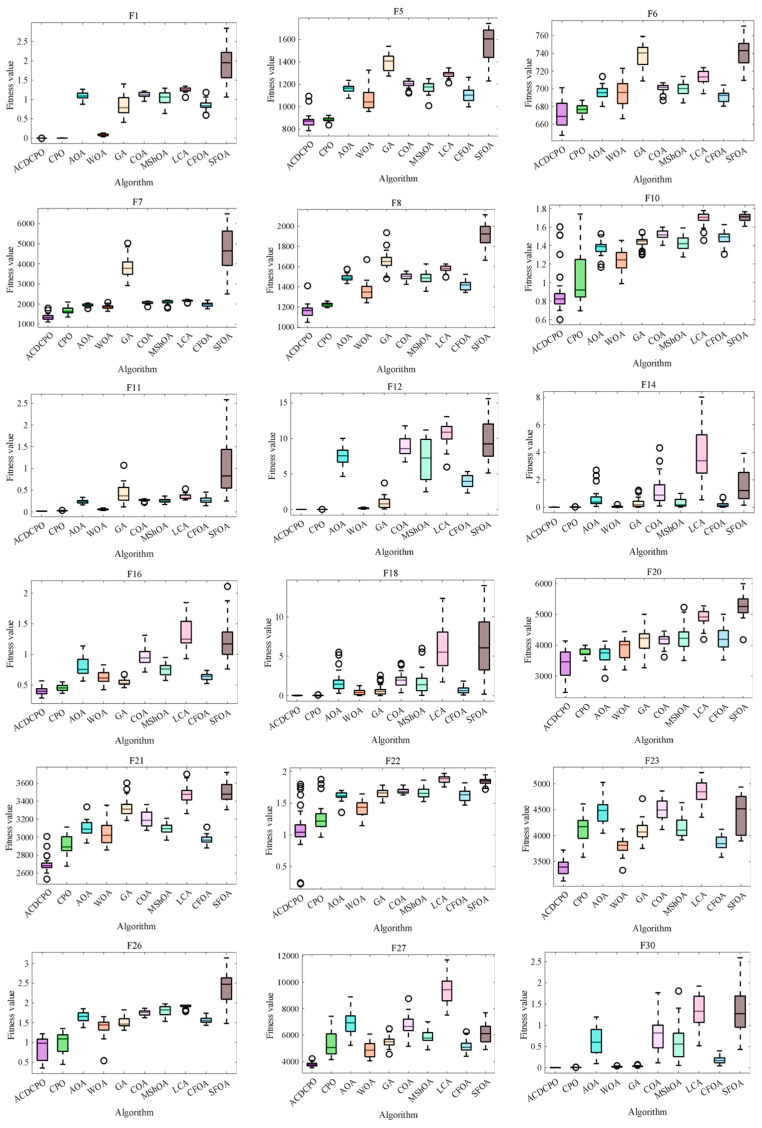
Box-plots of several CEC2017 functions.

Figure 4 shows the box plot comparison between ACDCPO and nine competing algorithms on selected CEC2017 benchmark functions. Overall, ACDCPO presents lower box positions, smaller median fitness values, and more compact distributions on most functions, indicating higher convergence accuracy and more stable optimization performance across multiple independent runs.

For simple functions, ACDCPO maintains a low and concentrated fitness distribution, showing its ability to quickly approach high-quality solutions. For complex functions such as *F*_12_, *F*_14_, and *F*_30_, several competitors, including SFOA and CFOA, exhibit wider distributions and higher fitness values, suggesting weaker stability and a greater tendency to fall into local optima. In contrast, ACDCPO keeps its fitness values within a lower and narrower range, demonstrating stronger global search capability and robustness.

In summary, the box plot results further confirm the effectiveness of the proposed multi-strategy improvement mechanism. The lower median reflects better convergence performance, while the narrower interquartile range indicates smaller fluctuations and stronger stability.

#### 3.2.4. Statistical Significance Analysis on CEC2017 Wilcoxon Test

Table 3 shows the results of the Wilcoxon test comparing ACDCPO with nine competitive algorithms. When compared against COA, MShOA, LCA, and SFOA, ACDCPO achieved *p*-values less than 0.05 across all 29 test functions, indicating a statistically significant performance advantage over these algorithms. In the comparison with the original CPO, only three functions yielded *p*-values greater than 0.05, while the remaining 26 functions showed *p*-values less than 0.05. This demonstrates that the improvements introduced in ACDCPO over the baseline CPO are statistically significant. The results show that the superior optimization performance of the ACDCPO algorithm is not accidental.

#### 3.2.5. Comprehensive Performance Evaluation Based on CEC2017

**Figure 5:**
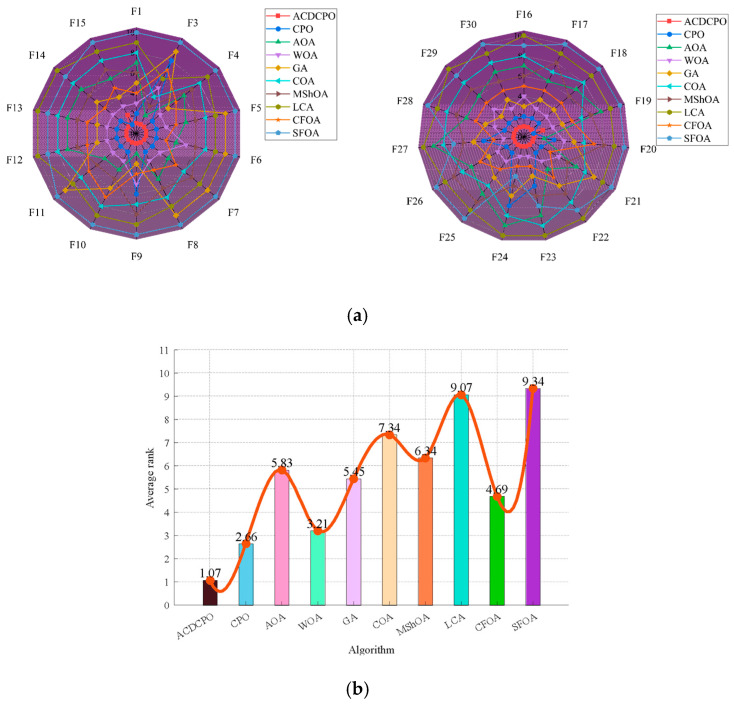
Experimental results on CEC2017. (**a**) Radar chart. (**b**) Average ranking chart.

Figure 5 shows the experimental comparison results on CEC2017. The ACDCPO algorithm has the smallest area in the radar map, ranks first on 27 functions, and ranks second on two functions, which demonstrates its superior stability and reliability. The ranking chart shows its average rank of 1.07, placing it first among all algorithms and reflecting outstanding comprehensive performance and stability.

### 3.3. Performance Evaluation and Analysis on CEC2022 Benchmark Functions

The CEC2022 dataset consists of 12 test functions: monotonic functions (*F*_1_) evaluate the local refinement capability and convergence efficiency of algorithms; simple multi-peak functions (*F*_2_–*F*_5_) contain numerous local optima to test global search capability; hybrid functions (*F*_6_–*F*_8_) combine multiple basic functions to assess complex multi-region search capability; composite functions (*F*_9_–*F*_12_) form complex landscapes by superimposing multiple functions to comprehensively evaluate overall optimization capability.

#### 3.3.1. Core Parameter Analysis on CEC2022 Benchmark Functions 

Table 4 presents the comparison results of the core parameters of the ACDCPO algorithm with other algorithms on the CEC2022 test functions (*F*_1_ to *F*_12_). Similar to CEC2017, the evaluation indicators used are min, std, and avg.

For the unimodal function, *F*_1_, ACDCPO demonstrates strong exploitation ability and convergence accuracy. It achieves the best minimum value of 1.17 × 10^3^, clearly outperforming CPO (1.38 × 10^4^), WOA (1.40 × 10^4^), and AOA (1.90 × 10^4^). In addition, ACDCPO obtains the lowest average value of 4.32 × 10^3^ and ranks first among all compared algorithms, indicating its superior optimization accuracy and stable performance over repeated runs.

For the simple multimodal functions, *F*_2_–*F*_5_, ACDCPO shows good overall performance. It ranks first on *F*_2_, *F*_3_, and *F*_4_, and second on *F*_5_, demonstrating strong global exploration ability and local optimum escaping capability. Taking *F*_2_ as an example, ACDCPO achieves the best minimum and average values, 4.46 × 10^2^ and 4.62 × 10^2^, respectively, which are better than those of the CPO, WOA, GA, and other algorithms. Although its standard deviation is not always the lowest on *F*_3_ and *F*_4_, its average performance and overall ranking remain highly competitive, suggesting a good balance between exploration and exploitation.

For the hybrid functions, *F*_6_–*F*_8_, ACDCPO ranks first on *F*_7_ and *F*_8_ and second on *F*_6_, indicating good adaptability to heterogeneous search spaces. On *F*_7_, ACDCPO obtains the lowest average value of 2.13 × 10^3^, outperforming CPO (2.31 × 10^3^) and most other comparison algorithms. Meanwhile, its relatively low standard deviation reflects stable search behavior across multiple independent runs.

For the composition functions, *F*_9_–*F*_12_, ACDCPO ranks first on *F*_9_, *F*_11_, and *F*_12_, and second on *F*_10_. On *F*_11_, ACDCPO achieves the best average value of 2.94 × 10^3^, while on F12, it also obtains the best average value of 3.00 × 10^3^. These results indicate that ACDCPO maintains high optimization accuracy and stable search performance on complex composition functions, further demonstrating its robustness in challenging optimization landscapes. In conclusion, ACDCPO combines high precision with high stability, yielding excellent comprehensive performance.

#### 3.3.2. Convergence Analysis on CEC2022 Benchmark Functions 

**Figure 6:**
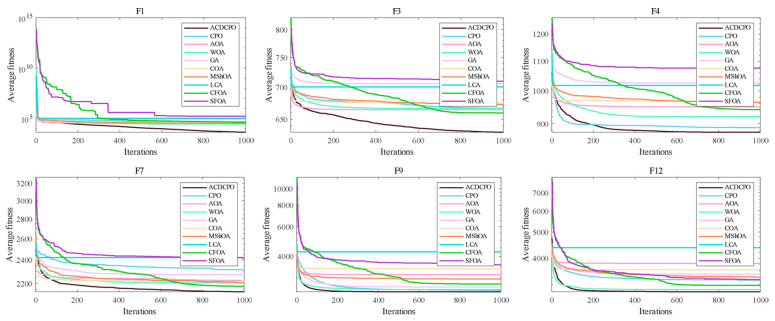
Convergence curves of the selected test functions in CEC2022.

Figure 6 presents the convergence curves of ACDCPO and compared algorithms on selected CEC2022 benchmark functions. On *F*_1_, *F*_3_, *F*_4_, and *F*_7_, ACDCPO declines rapidly in early iterations and stabilizes at a much lower fitness level than most counterparts, reflecting its excellent local exploitation capability, faster convergence speed, and higher convergence accuracy. For more complex functions such as *F*_9_ and *F*_12_, ACDCPO maintains a continuous downward trend and finally converges to a lower fitness range, demonstrating strong global search ability and robustness in complex landscapes. By contrast, some comparison algorithms suffer from slow convergence or premature stagnation: SFOA shows obvious optimization stagnation on *F*_7_, while CFOA remains at a relatively high fitness level on *F*_12_, indicating weaker optimization stability and higher vulnerability to local optima.

#### 3.3.3. Stability Analysis on CEC2022 Benchmark Functions

**Figure 7:**
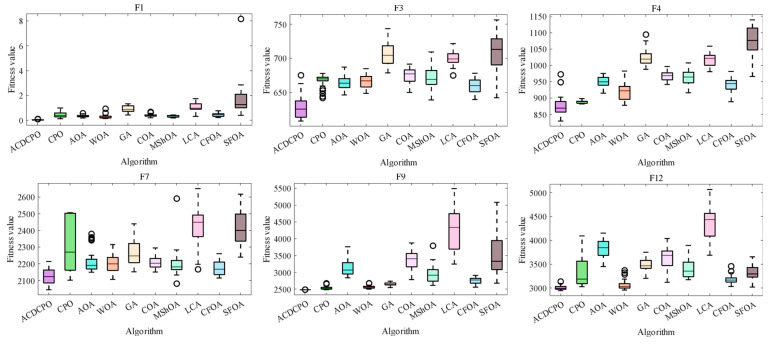
Box-plots for the selected CEC2022 benchmark functions.

Figure 7 presents the box plot comparison between ACDCPO and nine competing algorithms on selected CEC2022 benchmark functions. As shown, ACDCPO generally exhibits the lowest box positions and more compact distributions across the tested functions, indicating lower fitness values, better solution quality, and stronger optimization stability over multiple independent runs. Taking *F*_1_ as an example, the median fitness value of ACDCPO is close to zero and clearly lower than those of CPO, AOA, WOA, and other competitors, demonstrating its strong local exploitation ability and reliable convergence behavior.

For complex functions such as *F*_7_, *F*_9_, and *F*_12_, most competing algorithms show wider box distributions and higher median fitness values, reflecting larger performance fluctuations and weaker robustness. In contrast, ACDCPO maintains low and compact fitness distributions, suggesting that it can effectively reduce result dispersion while preserving high search accuracy and robustness on complex optimization landscapes.

#### 3.3.4. Statistical Significance Analysis on CEC2022 Wilcoxon Test

Analyzing the distribution of *p*-values in the Wilcoxon rank-sum test results in Table 5, compared with AOA, GA, COA, LCA, and SFOA, the *p*-values of ACDCPO on 12 test functions are less than 0.05, which verifies the statistical superiority of ACDCPO on these functions. Compared with WOA and MShOA, only one function has a *p*-value greater than 0.05, and the remaining 11 functions show significant superiority. This result strongly proves that the optimization performance of the ACDCPO algorithm is not determined by randomness, but has high consistency and reliability.

#### 3.3.5. Comprehensive Performance Evaluation Based on CEC2022

**Figure 8:**
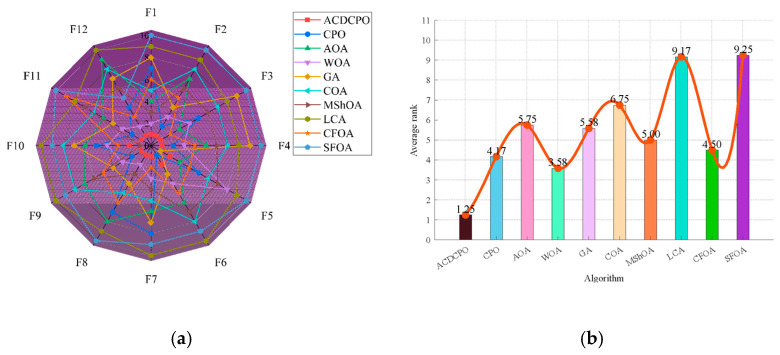
Experimental results on CEC2022. (**a**) Radar chart. (**b**) Average ranking chart.

Figure 8 shows the experimental comparison results on the CEC2022 dataset. In Figure 8a, the ACDCPO curve is closest to the center, reflecting its superior optimization accuracy and robustness across all functions. The average ranking chart quantifies this advantage, with ACDCPO ranking first at a mean value of 1.25 and remarkably outperforming all other algorithms. The second-ranked CPO has an average rank of 4.17, highlighting a substantial performance gap. These results confirm that ACDCPO possesses the best comprehensive optimization performance and competitiveness.

## 4. Engineering Experiments

Five engineering application problems are selected in this paper to verify the engineering application performance of the proposed algorithm, including four typical engineering design optimization problems and one moisture content prediction task of *Dendrobium huoshanense*. All the above problems feature varying degrees of nonlinearity, variable coupling, and complex constraints, which can comprehensively examine the optimization accuracy, stability, and robustness of the algorithm in practical engineering constrained optimization scenarios.

To ensure the fairness of the experimental comparison, all comparison algorithms adopt the same population size and number of independent runs for each engineering problem. Specifically, the population size is set to 30 for all four engineering problems, and each algorithm is run independently 30 times. Statistical indicators, including the best value, mean value, standard deviation (Std), and worst value, are calculated based on the results of multiple runs. With the above unified experimental settings, this paper comprehensively evaluates the solution capability of different algorithms in engineering optimization problems from the perspectives of optimization accuracy, search performance, and result stability.

### 4.1. Cantilever Beam Design Problem

The optimization objective of the cantilever beam design problem is to minimize the total weight of the cantilever beam, which consists of five segments with identical wall thickness and square hollow cross-sections. A vertical load is applied at the free end of the cantilever beam, and structural constraints are imposed to ensure the structural deformation meets design specifications.

**Figure 9:**
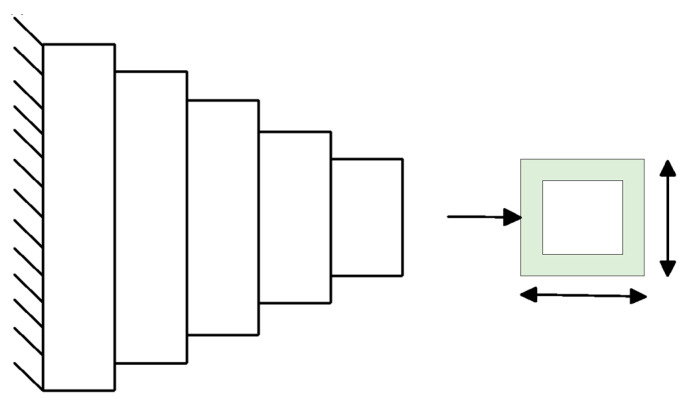
Cantilever beam design model.

This optimization model involves five decision variables denoted as (*x*_1_, *x*_2_, *x*_3_, *x*_4_, and *x*_5_), which correspond to the side lengths of the square cross-sections of the five beam segments from the fixed end to the free end [39]. Its structure is shown in Figure 9, and the specific model is given by Equations (37) and (38).

**Objective Function (Minimize):**

(37) $$f\left(x\right)=0.0624\left(x_{1}+x_{2}+x_{3}+x_{4}+x_{5}\right)$$

**Constraint Condition:**

(38) $$g\left(X\right)=\frac{61}{x_{1}^{3}}+\frac{37}{x_{2}^{3}}+\frac{19}{x_{3}^{3}}+\frac{7}{x_{4}^{3}}+\frac{1}{x_{5}^{3}}-1\leq 0$$

**Variable Bounds:**

0.01 ≤ *x_i_* ≤ 100, *i* = 1, 2, 3, 4, 5

where *f*(*x*) is the objective function representing the total weight of the cantilever beam, and *g*(*X*) denotes the deformation constraint. The optimization task is to find the optimal combination of side lengths for the five beam segments to minimize the total beam weight while complying with the structural deformation constraint.

Table 6 reports the statistical results of different algorithms on the cantilever beam design problem. For this specific engineering case, ACDCPO achieves the best values in all five statistical indicators. In particular, ACDCPO obtains the lowest best value of 1.3404, the lowest worst value of 1.3606, the smallest Std value of 0.0075, the lowest mean value of 1.3504, and the lowest median value of 1.3502. These results indicate that ACDCPO not only finds a high-quality optimal solution, but also maintains strong stability over repeated independent runs.

**Figure 10:**
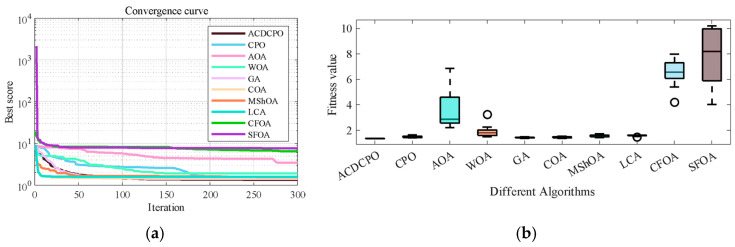
(**a**) Average convergence curve of cantilever beam design problem. (**b**) Box diagram of cantilever beam design problem.

The convergence curves in Figure 10a further show that ACDCPO converges rapidly during the early search stage and gradually stabilizes at a lower fitness level than the other algorithms. This suggests that the proposed improvement strategies are effective in accelerating convergence and improving solution accuracy. In addition, the box plot in Figure 10b shows that the distribution of ACDCPO is highly compact and located at the lowest fitness-value region, with no obvious outliers. This demonstrates that ACDCPO has better robustness and consistency in solving the cantilever beam design problem.

In summary, ACDCPO demonstrates superior convergence efficiency, higher solution precision, and stronger stability than the compared algorithms, making it more suitable for cantilever beam design tasks.

### 4.2. Pressure Vessel Design Problem

**Figure 11:**
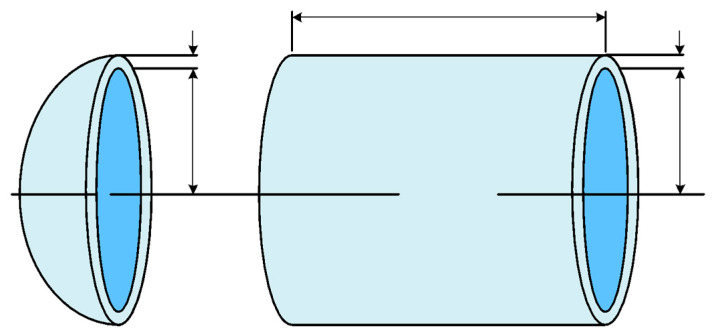
Pressure vessel design model.

The pressure vessel design problem is a representative constrained engineering optimization problem. The vessel consists of a cylindrical shell and two hemispherical heads. The optimization objective is to reduce the total manufacturing cost while satisfying the structural and volume requirements of the vessel [40]. The corresponding structure is illustrated in Figure 11.

Four design variables are considered in this problem: the shell thickness *T_s_*, the head thickness *T_h_*, the inner radius *R*, and the cylindrical section length *L*. These variables are denoted as *x*_1_, *x*_2_, *x*_3_, and *x*_4_, respectively, where *x* = (*x*_1_, *x*_2_, *x*_3_, *x*_4_) = (*T_s_*, *T_h_*, *R*, *L*). The detailed mathematical models are shown in Equations (32) and (33).

**Objective Function (Minimization):**

(39) $$f(x)=0.6224x_{1}x_{3}x_{4}+17781x_{2}x_{3}^{2}+3.1661x_{1}^{2}x_{4}+19.84x_{1}^{2}x_{3}$$

**Constraint Condition:**

(40) $$\begin{array}{l} s_{1}\left(x\right)=-x_{2}+0.0193x_{3}\leq 0\\ s_{2}\left(x\right)=-x_{1}+0.00954x_{3}\leq 0\\ s_{3}\left(x\right)=-\pi x_{3}^{2}x_{4}-\frac{4}{3}\pi x_{3}^{3}+1296000\leq 0\\ s_{4}\left(x\right)=x_{4}-240\leq 0 \end{array}$$

**Variable Bounds:**

0 ≤ *x*_1_ ≤ 99, 0 ≤ *x*_2_ ≤ 99, 10 ≤ *x*_3_ ≤ 200, 10 ≤ *x*_4_ ≤ 200

where *f*(*x*) represents the total manufacturing cost of the pressure vessel, and *s*_1_(*x*)–*s*_4_(*x*) denote the constraint functions related to shell thickness, head thickness, vessel volume, and cylinder length, respectively. The purpose of this optimization problem is to determine the optimal values of *T_s_*, *T_h_*, *R*, and *L*, so that the manufacturing cost is minimized while all design constraints are satisfied.

**Figure 12:**
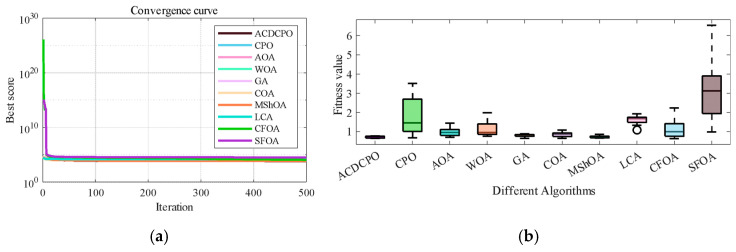
(**a**) Average convergence curve of pressure vessel design problem. (**b**) Box diagram of pressure vessel design problem.

Table 7 presents the statistical results of the pressure vessel design problem. Although ACDCPO does not achieve the best single-run best value of 6605.754354, it still ranks among the top with strong competitiveness. More critically, ACDCPO records the lowest mean (7160.757748), median (7104.061166), and standard deviation (454.8553237) values, reflecting better overall solution quality and superior stability and robustness across multiple independent runs. Figure 12a shows that ACDCPO keeps a steady downward trend and finally converges to a low fitness level. The box plot in Figure 12b further confirms its compact distribution in the low fitness-value region, meaning more consistent optimization results that are less affected by random fluctuations. Overall, ACDCPO exhibits remarkable comprehensive performance in average solution quality, convergence stability, and robustness for this problem. Though CFOA gains the single best result, the lower mean, median, and Std values of ACDCPO prove its more stable and reliable optimization performance in repeated runs.

### 4.3. Stepped Cone Pulley Problem

**Figure 13:**
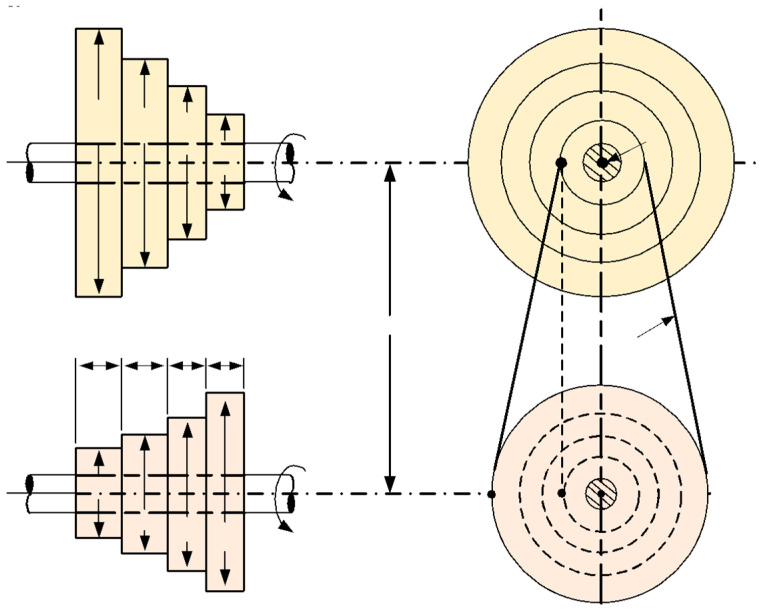
Stepped cone pulley problem model.

The stepped cone pulley problem is a constrained mechanical design optimization problem. Its main purpose is to reduce the total weight of a four-step cone pulley while ensuring that the pulley system satisfies the required power transmission performance. The design model contains five decision variables, including four pulley diameters and one common pulley width. These variables are expressed as *x* = (*d*_1_, *d*_2_, *d*_3_, *d*_4_, *w*), where *d*_1_, *d*_2_, *d*_3_, and *d*_4_ represent the diameters of the four pulley steps, and *w* denotes the pulley width [25]. Its structural layout is presented in Figure 13, followed by the corresponding mathematical formulation.

**Minimize:**

(41) $$f\left(x\right)=\rho w\left[d_{1}^{2}\left\{11+{\left(\frac{N_{1}}{N}\right)}^{2}\right\}+d_{2}^{2}\left\{1+{\left(\frac{N_{2}}{N}\right)}^{2}\right\}+d_{3}^{2}\left\{1+{\left(\frac{N_{3}}{N}\right)}^{2}\right\}+d_{4}^{2}\left\{1+{\left(\frac{N_{4}}{N}\right)}^{2}\right\}\right]$$

where *ρ* denotes the material density and *w* is related to the pulley width. To ensure the same belt length under different speed ratios, three equality constraints are introduced:

$$h_{1}\left(x\right)=K_{1}-K_{2}=0,h_{2}\left(x\right)=K_{1}-K_{3}=0,h_{3}\left(x\right)=K_{1}-K_{4}=0$$

In addition, the pulley system must satisfy nonlinear constraints related to the transmission ratio and power transmission capacity. These constraints can be expressed as:

$$\begin{array}{cc} R_{i}\geq 2,i=1,2,3,4 & P_{i} \end{array}\geq 0.75\times 745.6998,i=1,2,3,4$$

where *K_i_*, *R_i_*, and *P_i_* are calculated as:

(42) $$K_{i}=\frac{\pi d_{i}}{2}\left(1+\frac{N_{i}}{N}\right)+\frac{{\left(\frac{N_{i}}{N}-1\right)}^{2}}{4a}+2a,i=\left(1,2,3,4\right)\;R_{i}=\exp\left[\mu \left\{\pi -2\sin^{-1}\left\{\left(\frac{N_{i}}{N}-1\right)\frac{d_{i}}{2a}\right\}\right\}\right],i=\left(1,2,3,4\right)\;P_{i}=stw\left(1-R_{i}\right)\frac{\pi d_{i}N_{i}}{60},i=\left(1,2,3,4\right)$$

The relevant constant parameters are set as follows:

$$\rho =7200kg/m^{3},a=3m,\mu =0.35,s=1.75MPa,t=8mm$$

The boundary constraints of the design variables are given by:

(43) $$0\leq d_{1},d_{2}\leq 60,0\leq d_{3},d_{4},w\leq 90$$

In this problem, the objective function reflects the total weight of the pulley structure, while the equality and inequality constraints ensure the consistency of belt length, the required transmission ratio, and the minimum power transmission capacity. Therefore, this benchmark can be used to evaluate the ability of the proposed algorithm to solve nonlinear constrained engineering optimization problems with multiple coupled design variables.

Table 8 presents the statistical results for the stepped cone pulley design problem. For this engineering optimization problem, the proposed ACDCPO algorithm demonstrates strong comprehensive performance. Although ACDCPO does not obtain the best single-run value, its best fitness value of 6443.29855 remains highly competitive and is close to the best result achieved by WOA. More importantly, ACDCPO achieves the lowest worst value, standard deviation, and mean value among all compared algorithms, with values of 677,038.1808, 217,016.9128, and 262,749.5508, respectively. These results indicate that ACDCPO can maintain better overall solution quality and significantly reduce performance fluctuations across multiple independent runs.

**Figure 14:**
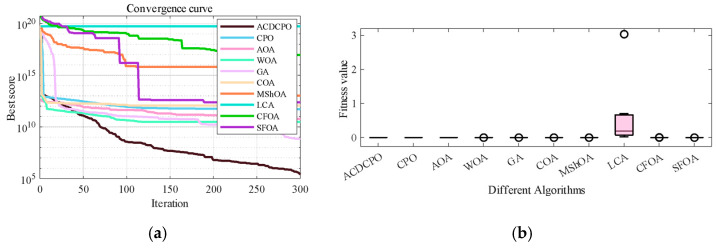
(**a**) Average convergence curve of stepped cone pulley problem. (**b**) Box diagram of stepped cone pulley problem.

The convergence curves in Figure 14a further show that ACDCPO maintains a steady downward trend throughout the iteration process and finally converges to a lower fitness level than most competing algorithms within 300 iterations. This demonstrates its strong search efficiency and convergence stability. In addition, the box plot in Figure 14b shows that the fitness distribution of ACDCPO is concentrated in a low-value region, further confirming its stable and reliable optimization performance.

### 4.4. Optimization Design Problem for Industrial Refrigeration Systems

Optimizing industrial refrigeration systems presents a complex engineering design challenge, aiming to balance multiple objectives such as cooling performance, energy efficiency, and reliability to achieve a highly efficient, low-consumption, and stable system. This problem involves 14 design variables and 15 restrictions, with the corresponding mathematical model presented below [41]:

The decision vector is defined as:

(44) $$x=\left[x_{1},x_{2},x_{3},x_{4},x_{5},x_{6},x_{7},x_{8},x_{9},x_{10},x_{11},x_{12},x_{13},x_{14}\right]$$

The objective function of the industrial refrigeration system is formulated as follows:

(45) $$\begin{array}{l} f\left(x\right)=63098.88x_{2}x_{4}x_{12}+5441.5x_{2}^{2}x_{12}+115055.5x_{2}^{1.664}x_{6}+6172.27x_{2}^{2}x_{6}+63098.88x_{1}x_{3}x_{11}\\ +5441.5x_{1}^{2}x_{11}+115055.5x_{1}^{1.664}x_{5}+6172.27x_{1}^{5}+140.53x_{1}x_{11}+281.29x_{3}x_{11}+70.26x_{1}^{2}\\ +281.29x_{1}x_{3}+281.29x_{3}^{2}+14437x_{8}^{1.8812}x_{12}^{0.3424}x_{10}x_{14}^{-1}x_{7}x_{9}^{-1}+20470x_{7}^{2.893}x_{11}^{0.316}x_{1}^{2} \end{array}$$

The system is subject to fifteen nonlinear constraints, which can be expressed as:

(46) $$\begin{array}{l} y_{1}\left(x\right)=1.524x_{7}^{-1}\leq 1\\ y_{2}\left(x\right)=1.524x_{8}^{-1}\leq 1\\ y_{3}\left(x\right)=0.07789x_{1}-2x_{7}^{-3}x_{9}\leq 1\\ y_{4}\left(x\right)=7.05305x_{9}^{-1}x_{1}^{2}x_{10}x_{8}^{-1}x_{2}^{-1}x_{14}^{-1}-1\leq 0\\ y_{5}\left(x\right)=0.0833x_{13}^{-1}x_{14}-1\leq 0\\ y_{6}\left(x\right)=47.136x_{2}^{0.333}x_{10}^{-1}x_{12}-1.333x_{8}x_{13}^{2.1195}+62.08x_{13}^{2.1195}x_{12}^{-1}x_{8}^{0.2}x_{10}^{-1}-1\leq 0\\ y_{7}\left(x\right)=0.04771x_{10}x_{8}^{1.8812}x_{12}^{0.3424}-1\leq 0\\ y_{8}\left(x\right)=0.0488x_{9}x_{7}^{1.893}x_{11}^{0.316}-1\leq 0\\ y_{9}\left(x\right)=0.0099x_{1}x_{3}^{-1}-1\leq 0\\ y_{10}\left(x\right)=0.0193x_{2}x_{4}^{-1}-1\leq 0\\ y_{11}\left(x\right)=0.0298x_{1}x_{5}^{-1}-1\leq 0\\ y_{12}\left(x\right)=0.056x_{2}x_{6}^{-1}-1\leq 0\\ y_{13}\left(x\right)=2x_{9}^{-1}-1\leq 0\\ y_{14}\left(x\right)=2x_{10}^{-1}-1\leq 0\\ y_{15}\left(x\right)=x_{12}x_{11}^{-1}-1\leq 0 \end{array}$$

where the constraint functions describe the design requirements related to refrigeration capacity, equipment operation, system matching, and feasible operating ranges. The boundary range of each decision variable is given by:

$$0.001\leq x_{i}\leq 5,i=1,\ldots ,14$$

According to the statistical results in Table 9, although ACDCPO does not obtain the best single-run optimal value, it achieves the lowest worst value, standard deviation, and mean value among all compared algorithms. This indicates that ACDCPO has better average solution quality, smaller performance fluctuations, and stronger robustness across multiple independent runs.

**Figure 15:**
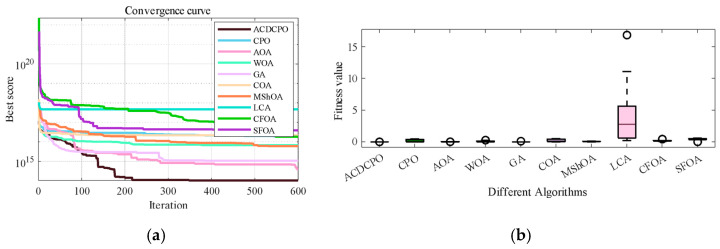
(**a**) Average convergence curve of the optimization design problem for industrial refrigeration systems. (**b**) Box diagram of the optimization design problem for industrial refrigeration systems.

As shown in the convergence curve in Figure 15a, the fitness value of ACDCPO decreases rapidly in the early iteration stage, continues to improve during the search process, and finally converges to a relatively low fitness level. This demonstrates its effective global search capability and stable convergence behavior. The box plot in Figure 15b further shows that the fitness distribution of ACDCPO is concentrated in the low-value region, indicating more stable and reliable optimization performance. Therefore, ACDCPO provides a reliable and efficient method for the optimal design of industrial refrigeration systems.

### 4.5. Moisture Content Prediction of Dendrobium huoshanense Based on the ACDCPO Algorithm

Moisture content serves as a key indicator for assessing the quality of *Dendrobium huoshanense*, which directly affects its storage shelf life, stability of active ingredients, and risk of microbial growth. Accurate prediction of moisture content is of great practical significance for the standardized storage, quality control, and efficacy guarantee of *Dendrobium huoshanense*. Traditional detection methods are time-consuming and destructive. Although the BP neural network can realize moisture content prediction, it is prone to falling into local optimal solutions, and its prediction accuracy and generalization ability are significantly affected by initial weights and biases, making high-precision prediction difficult. Therefore, the ACDCPO algorithm is adopted to determine the optimal initial weight *w* and bias *b* of the BP neural network [42], so as to enhance the network’s parameter optimization ability, and improve the model convergence speed and prediction stability.

#### 4.5.1. Data Processing

**Figure 16:**
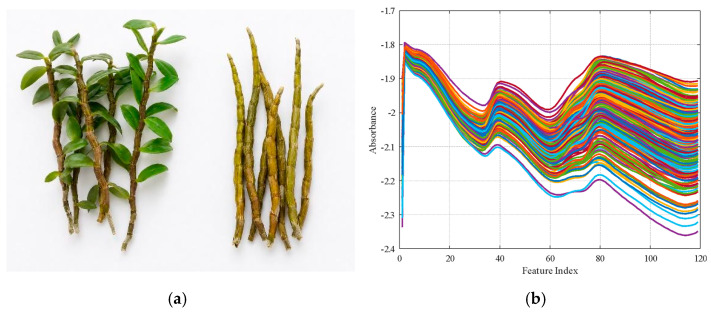
(**a**) Physical samples of *Dendrobium huoshanense*. (**b**) Near-infrared spectral curves.

The experimental data used in this study were obtained from measured near-infrared spectra of *Dendrobium huoshanense*. A total of 275 valid samples were collected. Each sample contains 119 spectral feature variables and one measured moisture content label. The spectral variables were used as the model inputs, while the measured moisture content was used as the model output target. Figure 16a shows the physical samples of *Dendrobium huoshanense*, and Figure 16b presents the near-infrared spectral curves of the samples.

To reduce the interference of random data splitting on experimental results and ensure a consistent evaluation benchmark for all comparative models, a dataset splitting strategy with a fixed random seed is adopted in this study. First, a fixed random seed is set to shuffle all 275 samples randomly. Afterwards, the samples are divided into a training set (220 samples) and a test set (55 samples) at an 8:2 ratio. This splitting scheme remains fixed across all comparative experiments, and all BP models optimized by different optimization algorithms share exactly the same training and test sets. This design guarantees that performance discrepancies among various models stem solely from the optimization capability of the algorithms rather than the randomness in data partitioning. It effectively reduces experimental random errors and improves the reproducibility of results as well as the reliability of conclusions.

To eliminate the impacts of differences in dimension and numerical range of different spectral variables on model training, the Min—Max normalization method is utilized to scale the inputs and outputs of the training set into the interval [0, 1], as shown in Equation (47):

(47) $${\tilde{p}}_{i,j}=\frac{p_{i,j}-p_{i,\min}}{p_{i,\max}-p_{i,\min}},{\tilde{t}}_{j}=\frac{t_{j}-t_{\min}}{t_{\max}-t_{\min}}$$

where $p_{i,j}$ denotes the original feature value; $p_{i,\min}$ and $p_{i,\max}$ represent the minimum and maximum values of the *i*-th feature in the training set, respectively; and ${\tilde{p}}_{i,j}$ stands for the normalized feature value. *t_j_* is the original moisture content; $t_{\min}$ and $t_{\max}$ are the minimum and maximum moisture content values of the training set, respectively; and ${\tilde{t}}_{j}$ refers to the normalized moisture content value.

#### 4.5.2. Construction of the ACDCPO-BP Prediction Model

To establish the nonlinear mapping relationship between near-infrared spectral features and the moisture content of *Dendrobium huoshanense*, a three-layer BP neural network is adopted in this paper. The network structure consists of an input layer, a hidden layer, and an output layer. The number of neurons in the input layer is *p* = 119, the number of hidden layer neurons is *h* = 5, and the number of output layer neurons is *q* = 1. The total dimension of the optimization problem is calculated as *D_all_* = *p* × *h* + *h* + *h* × 1 + 1 = 606.

1. Output of the Hidden Layer

(48) $$h_{j}=\varphi \left(W_{1}{\tilde{p}}_{j}+b_{1}\right)$$

In Equation (48), ${\tilde{p}}_{j}$ is the normalized input vector of the *j*-th sample; *W*_1_ denotes the weight matrix from the input layer to the hidden layer; *b*_1_ is the bias vector of the hidden layer; *φ* represents the activation function of the hidden layer; and *h_j_* is the output vector of the hidden layer.

2. Output of the Output Layer

(49) $${\hat{t}}_{j}=W_{2}h_{j}+b_{2}$$

In Equation (49), *W*_2_ denotes the weight matrix from the hidden layer to the output layer; *b*_2_ is the bias of the output layer; and ${\hat{t}}_{j}$ represents the predicted normalized moisture content. ACDCPO flattens all weights and biases of the BP network into a one-dimensional parameter vector, which serves as the candidate solution for the optimization problem. The parameter vector *θ* to be optimized is defined as:

(50) $$\theta ={\left[vec\left(W_{1}\right),b_{1}^{T},vec\left(W_{2}\right),b_{2}\right]}^{T}$$

where *vec*(∙) denotes the matrix vectorization operation, which reshapes a matrix into a one-dimensional vector column by column.

During the iterative optimization process of ACDCPO, the mean squared error of the training set is adopted as the fitness function. For any candidate parameter vector *θ*, the fitness value is defined as:

(51) $$F\left(\theta \right)=\frac{1}{M}{\sum_{j=1}^{M}\left({\tilde{t}}_{j}-{\hat{t}}_{j}\left(\theta \right)\right)}^{2}$$

In Equation (51), *M* is the number of samples in the training set; ${\tilde{t}}_{j}$ is the measured normalized moisture content of the *j*-th sample; and ${\hat{t}}_{j}\left(\theta \right)$ is the normalized predicted value of the network corresponding to the parameter vector *θ*.

After the optimal parameter vector $\theta^{*}$ is obtained through ACDCPO iterative optimization, it is restored to weight matrices and bias vectors, which are assigned to the BP network as initial parameters. Then, the fine-tuning training of the network is completed via BP backpropagation.

#### 4.5.3. Experimental Setup

**Figure 17:**
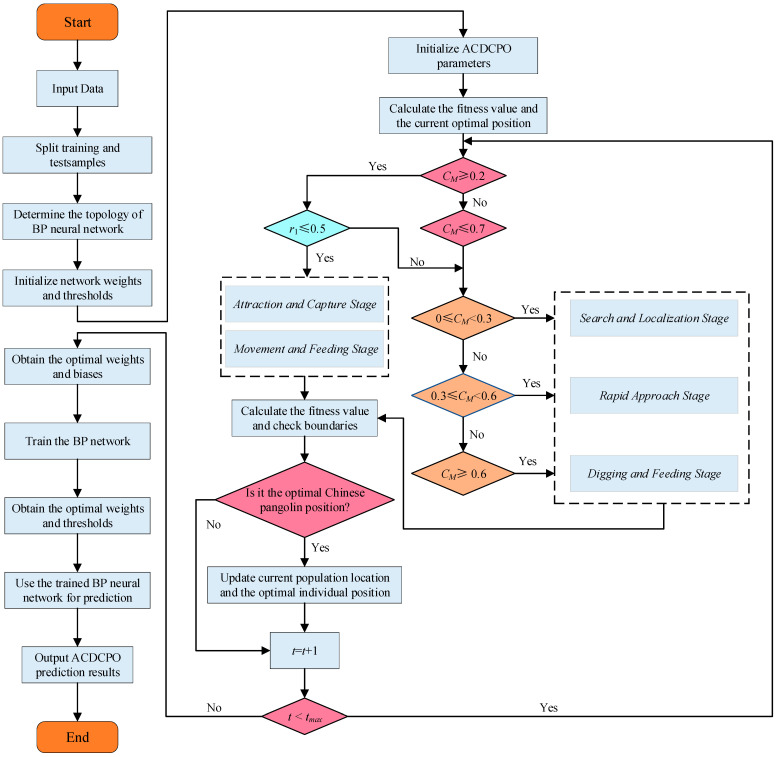
Flowchart of the ACDCPO-BP model.

For all algorithms, the maximum number of iterations was set to 100, and the population size was set to 50. To ensure the accuracy and reliability of the test results, each algorithm was independently run 20 times, and the average value was taken as the final experimental result. The overall optimization flow of the BP network is illustrated in Figure 17.

#### 4.5.4. Comparison with Other Benchmark Models

To comprehensively evaluate the prediction accuracy and stability of the ACDCPO-optimized BP model, five evaluation metrics were adopted in this study, including four error-based indicators, namely MAE, MAPE, MSE, and RMSE, and the coefficient of determination R^2^ [43]. The error-based indicators measure the deviation between the predicted and measured values, where smaller values indicate better prediction performance. In contrast, R^2^ reflects the degree of agreement between the predicted and measured values, with a larger value indicating better fitting performance. For compact and unified presentation, the values of the five indicators in Table 10 are reported after scaling, with the corresponding scale factors of MAE (×10^1^), MAPE (×10^0^), MSE (×10^3^), RMSE (×10^1^), and R^2^ (×10^−1^), respectively.

Table 10 presents the comparison results of the five evaluation metrics for different BP-based prediction models. The results show that ACDCPO-BP achieves competitive prediction performance among the compared models and improves the parameter optimization ability of the BP neural network. Compared with the original CPO-BP model, ACDCPO-BP obtains improvements in all evaluation metrics. Specifically, the MAE decreases from 31.183 to 28.735, corresponding to a reduction of 7.85%. The MAPE decreases from 8.712% to 8.0408%, with a reduction of 7.71%. The MSE decreases from 1540.1 to 1351.6, corresponding to a reduction of 12.24%, while the RMSE decreases from 38.816 to 36.781, with a reduction of 5.24%. In addition, R^2^ increases from 89.126% to 91.211%, indicating improved fitting ability.

## 5. Summary of Work and Future Prospects

### 5.1. Summary of Work

Addressing the inherent defects of the Chinese Pangolin Optimization (CPO) algorithm, this paper proposes an improved algorithm incorporating hybrid multi-strategies, named the ACDCPO algorithm. The proposed method integrates a boundary-adaptive contraction initialization strategy, a Cauchy inverse cumulative distribution mutation mechanism, and dynamic opposition-based learning. These strategies effectively enhance population diversity and improve the balance between the algorithm’s capacity for global exploration and local refinement.

The results on the CEC 2017 and CEC 2022 benchmarks show that ACDCPO delivers leading or competitive performance on most functions, achieving the best overall rank. Specifically, ACDCPO demonstrates faster convergence, improved solution precision, and greater robustness relative to other methods. Statistical analyses, including convergence curves and box plots, further corroborate that ACDCPO not only rapidly approaches the global optimum region but also yields results with minimal fluctuations. The results confirm the superior robustness of the proposed algorithm over the original CPO and other competitors.

In the four engineering optimization problems, the ACDCPO algorithm can achieve lower mean and standard deviation in all cases. These results demonstrate its strong solution capability and stability when addressing practical engineering problems with complex constraints and multiple variables. Moreover, the algorithm consistently produces high-quality feasible solutions, indicating its significant potential for engineering applications.

The ACDCPO-BP model had the lowest MAE, MAPE, MSE, and RMSE values, and the highest coefficient of determination (R^2^) in the task of predicting the water content of *Dendrobium huoshanense* based on the BP network optimized by ACDCPO. This indicates that ACDCPO can significantly enhance the predictive performance and generalization capability of BP neural networks, verifying its application potential in machine learning parameter optimization.

### 5.2. Future Prospects

Although the ACDCPO algorithm delivers strong optimization results, further investigation and refinement are still needed. Future work can be conducted based on the following aspects:

(1) Research on Parameter Adaptive Mechanism

Some parameters in this paper are still set as fixed values, and different optimization problems may correspond to different optimal parameter combinations. In the future, an adaptive parameter adjustment mechanism or learning strategy can be introduced to enable the algorithm to dynamically adjust key parameters according to the search state, thereby further improving the optimization performance.

(2) Integration with Deep Learning Models

This paper only verifies the optimization capability of ACDCPO in BP neural networks. In the future, it can be further applied to deep neural networks, Transformer models, feature selection, and other tasks to improve the training efficiency and prediction performance of complex models.

## Figures and Tables

**Table 1:** Classification and evaluation purposes of CEC2017 benchmark functions.

Function Category	Representative Functions	Main Characteristics	Evaluation Purpose
Unimodal functions	F1 and F3	Only one global optimum; no local optima; relatively simple landscape	Evaluate exploitation ability, convergence speed, and convergence accuracy
Simple multimodal functions	F4–F10	Multiple local optima; prone to local stagnation	Evaluate global exploration ability and ability to escape local optima
Hybrid functions	F11–F20	Combination of different basic functions in different variable subcomponents; heterogeneous landscape	Evaluate adaptability, robustness, and exploration–exploitation balance
Composition functions	F21–F30	Nonlinear combination of multiple functions with shifting, rotation, and weighting; highly complex landscape	Evaluate comprehensive optimization ability, stability, and resistance to premature convergence

**Table 2:** Test results for CEC2017 test functions at 50.

Function	Type	ACDCPO	CPO	AOA	WOA	GA	COA	MShOA	LCA	CFOA	SFOA
*F*_1_	min	5.90 × 10^6^	1.20 × 10^7^	8.79 × 10^10^	3.71 × 10^9^	4.10 × 10^10^	9.55 × 10^10^	6.39 × 10^10^	1.06 × 10^11^	5.98 × 10^10^	1.06 × 10^11^
	std	1.05 × 10^7^	6.06 × 10^7^	1.01 × 10^10^	2.43 × 10^9^	2.63 × 10^10^	6.78 × 10^9^	1.62 × 10^10^	5.93 × 10^9^	1.18 × 10^10^	4.24 × 10^10^
	avg	1.72 × 10^7^	7.47 × 10^7^	1.10 × 10^11^	8.04 × 10^9^	8.67 × 10^10^	1.12 × 10^11^	1.04 × 10^11^	1.26 × 10^11^	8.55 × 10^10^	1.93 × 10^11^
	rank	1	2	7	3	5	8	6	9	4	10
*F*_3_	min	7.32 × 10^4^	1.54 × 10^5^	1.43 × 10^5^	1.69 × 10^5^	2.37 × 10^5^	1.61 × 10^5^	1.34 × 10^5^	2.05 × 10^5^	1.90 × 10^5^	3.40 × 10^5^
	std	2.28 × 10^4^	8.63 × 10^4^	1.47 × 10^4^	7.45 × 10^4^	9.90 × 10^4^	2.74 × 10^4^	2.17 × 10^4^	1.57 × 10^5^	1.03 × 10^5^	4.62 × 10^6^
	avg	1.27 × 10^5^	3.51 × 10^5^	1.74 × 10^5^	2.53 × 10^5^	4.12 × 10^5^	1.98 × 10^5^	1.86 × 10^5^	3.27 × 10^5^	3.38 × 10^5^	2.64 × 10^6^
	rank	1	8	2	5	9	4	3	6	7	10
*F*_4_	min	5.69 × 10^2^	6.14 × 10^2^	2.11 × 10^4^	1.54 × 10^3^	3.25 × 10^3^	3.21 × 10^4^	2.27 × 10^4^	3.40 × 10^4^	1.11 × 10^4^	3.55 × 10^4^
	std	5.27 × 10^1^	6.01 × 10^1^	7.78 × 10^3^	6.69 × 10^2^	4.79 × 10^3^	4.70 × 10^3^	8.31 × 10^3^	5.87 × 10^3^	4.66 × 10^3^	3.07 × 10^4^
	avg	6.73 × 10^2^	7.32 × 10^2^	3.60 × 10^4^	2.54 × 10^3^	9.72 × 10^3^	4.03 × 10^4^	3.79 × 10^4^	4.93 × 10^4^	2.15 × 10^4^	8.17 × 10^4^
	rank	1	2	6	3	4	8	7	9	5	10
*F*_5_	min	7.85 × 10^2^	8.37 × 10^2^	1.08 × 10^3^	9.57 × 10^2^	1.27 × 10^3^	1.12 × 10^3^	1.01 × 10^3^	1.21 × 10^3^	9.97 × 10^2^	1.23 × 10^3^
	std	6.49 × 10^1^	1.74 × 10^1^	3.80 × 10^1^	9.08 × 10^1^	8.07 × 10^1^	3.02 × 10^1^	5.31 × 10^1^	2.90 × 10^1^	5.75 × 10^1^	1.41 × 10^2^
	avg	8.70 × 10^2^	8.84 × 10^2^	1.16 × 10^3^	1.07 × 10^3^	1.40 × 10^3^	1.20 × 10^3^	1.17 × 10^3^	1.29 × 10^3^	1.11 × 10^3^	1.56 × 10^3^
	rank	1	2	5	3	9	7	6	8	4	10
*F*_6_	min	6.48 × 10^2^	6.65 × 10^2^	6.80 × 10^2^	6.66 × 10^2^	7.09 × 10^2^	6.87 × 10^2^	6.84 × 10^2^	6.95 × 10^2^	6.81 × 10^2^	7.09 × 10^2^
	std	1.58 × 10^1^	5.89 × 10^0^	7.00 × 10^0^	1.42 × 10^1^	1.36 × 10^1^	4.28 × 10^0^	7.74 × 10^0^	6.68 × 10^0^	6.16 × 10^0^	1.48 × 10^1^
	avg	6.71 × 10^2^	6.77 × 10^2^	6.95 × 10^2^	6.95 × 10^2^	7.38 × 10^2^	7.01 × 10^2^	7.00 × 10^2^	7.14 × 10^2^	6.91 × 10^2^	7.42 × 10^2^
	rank	1	2	4	5	9	7	6	8	4	10
*F*_7_	min	1.11 × 10^3^	1.35 × 10^3^	1.77 × 10^3^	1.64 × 10^3^	2.92 × 10^3^	1.87 × 10^3^	1.80 × 10^3^	2.05 × 10^3^	1.76 × 10^3^	2.50 × 10^3^
	std	1.57 × 10^2^	1.86 × 10^2^	7.49 × 10^1^	9.81 × 10^1^	4.99 × 10^2^	7.10 × 10^1^	9.70 × 10^1^	4.13 × 10^1^	1.20 × 10^2^	1.13 × 10^3^
	avg	1.35 × 10^3^	1.67 × 10^3^	1.94 × 10^3^	1.86 × 10^3^	3.79 × 10^3^	2.06 × 10^3^	2.09 × 10^3^	2.17 × 10^3^	1.97 × 10^3^	4.73 × 10^3^
	rank	1	2	4	3	9	6	7	8	5	10
*F*_8_	min	1.05 × 10^3^	1.19 × 10^3^	1.43 × 10^3^	1.24 × 10^3^	1.48 × 10^3^	1.42 × 10^3^	1.36 × 10^3^	1.50 × 10^3^	1.34 × 10^3^	1.66 × 10^3^
	std	6.40 × 10^1^	1.67 × 10^1^	3.62 × 10^1^	8.50 × 10^1^	9.25 × 10^1^	3.55 × 10^1^	5.81 × 10^1^	3.31 × 10^1^	4.91 × 10^1^	1.13 × 10^2^
	avg	1.16 × 10^3^	1.22 × 10^3^	1.49 × 10^3^	1.36 × 10^3^	1.65 × 10^3^	1.50 × 10^3^	1.49 × 10^3^	1.58 × 10^3^	1.42 × 10^3^	1.92 × 10^3^
	rank	1	2	5	3	9	7	6	8	4	10
*F*_9_	min	9.78 × 10^3^	1.55 × 10^4^	2.14 × 10^4^	2.15 × 10^4^	1.75 × 10^4^	2.66 × 10^4^	2.81 × 10^4^	4.35 × 10^4^	2.15 × 10^4^	4.88 × 10^4^
	std	8.39 × 10^3^	1.04 × 10^4^	3.95 × 10^3^	1.02 × 10^4^	6.85 × 10^3^	4.72 × 10^3^	5.10 × 10^3^	3.21 × 10^3^	7.63 × 10^3^	1.74 × 10^4^
	avg	2.90 × 10^4^	3.49 × 10^4^	2.93 × 10^4^	3.36 × 10^4^	3.14 × 10^4^	3.64 × 10^4^	3.76 × 10^4^	4.91 × 10^4^	3.34 × 10^4^	9.36 × 10^4^
	rank	1	6	2	5	3	7	8	9	4	10
*F*_10_	min	6.02 × 10^3^	6.94 × 10^3^	1.17 × 10^4^	9.90 × 10^3^	1.30 × 10^4^	1.40 × 10^4^	1.28 × 10^4^	1.46 × 10^4^	1.31 × 10^4^	1.61 × 10^4^
	std	2.49 × 10^3^	3.57 × 10^3^	8.31 × 10^2^	1.04 × 10^3^	6.34 × 10^2^	5.24 × 10^2^	7.55 × 10^2^	7.46 × 10^2^	8.15 × 10^2^	4.21 × 10^2^
	avg	9.03 × 10^3^	1.08 × 10^4^	1.38 × 10^4^	1.24 × 10^4^	1.43 × 10^4^	1.51 × 10^4^	1.42 × 10^4^	1.69 × 10^4^	1.48 × 10^4^	1.70 × 10^4^
	rank	1	2	4	3	6	8	5	9	7	10
*F*_11_	min	1.44 × 10^3^	1.43 × 10^3^	1.61 × 10^4^	3.39 × 10^3^	1.14 × 10^4^	2.19 × 10^4^	1.69 × 10^4^	2.77 × 10^4^	1.43 × 10^4^	2.51 × 10^4^
	std	1.37 × 10^2^	5.07 × 10^2^	4.00 × 10^3^	1.31 × 10^3^	2.09 × 10^4^	2.01 × 10^3^	4.87 × 10^3^	5.90 × 10^3^	6.99 × 10^3^	6.69 × 10^4^
	avg	1.76 × 10^3^	2.58 × 10^3^	2.31 × 10^4^	5.62 × 10^3^	4.20 × 10^4^	2.71 × 10^4^	2.63 × 10^4^	3.45 × 10^4^	2.63 × 10^4^	1.04 × 10^5^
	rank	1	2	4	3	9	7	5	8	6	10
*F*_12_	min	1.29 × 10^7^	1.35 × 10^7^	4.65 × 10^10^	5.31 × 10^8^	5.86 × 10^8^	6.71 × 10^10^	2.51 × 10^10^	6.00 × 10^10^	2.31 × 10^10^	5.14 × 10^10^
	std	6.36 × 10^7^	8.58 × 10^7^	1.29 × 10^10^	6.52 × 10^8^	8.42 × 10^9^	1.36 × 10^10^	2.86 × 10^10^	1.66 × 10^10^	8.21 × 10^9^	2.89 × 10^10^
	avg	1.23 × 10^8^	1.26 × 10^8^	7.45 × 10^10^	1.77 × 10^9^	9.63 × 10^9^	8.82 × 10^10^	7.01 × 10^10^	1.07 × 10^11^	3.94 × 10^10^	9.82 × 10^10^
	rank	1	2	7	3	4	8	6	10	5	9
*F*_13_	min	8.78 × 10^4^	2.58 × 10^4^	1.86 × 10^10^	5.73 × 10^6^	6.10 × 10^6^	1.76 × 10^10^	5.11 × 10^9^	3.37 × 10^10^	6.02 × 10^9^	9.31 × 10^9^
	std	1.10 × 10^5^	5.50 × 10^5^	1.35 × 10^10^	9.68 × 10^7^	8.31 × 10^8^	1.52 × 10^10^	2.24 × 10^10^	1.60 × 10^10^	5.95 × 10^9^	1.99 × 10^10^
	avg	2.20 × 10^5^	2.30 × 10^5^	4.04 × 10^10^	1.17 × 10^8^	6.28 × 10^8^	4.87 × 10^10^	3.76 × 10^10^	7.61 × 10^10^	1.46 × 10^10^	4.95 × 10^10^
	rank	1	2	7	3	4	8	6	10	5	9
*F*_14_	min	1.49 × 10^5^	7.28 × 10^4^	7.57 × 10^6^	5.54 × 10^5^	1.15 × 10^6^	9.79 × 10^6^	2.51 × 10^6^	5.41 × 10^7^	1.73 × 10^6^	1.55 × 10^7^
	std	5.16 × 10^5^	1.22 × 10^6^	6.37 × 10^7^	4.40 × 10^6^	3.21 × 10^7^	1.01 × 10^8^	2.84 × 10^7^	1.89 × 10^8^	1.73 × 10^7^	1.11 × 10^8^
	avg	8.03 × 10^5^	1.48 × 10^6^	6.11 × 10^7^	4.80 × 10^6^	3.04 × 10^7^	1.19 × 10^8^	3.13 × 10^7^	3.66 × 10^8^	1.95 × 10^7^	1.58 × 10^8^
	rank	1	2	7	3	5	8	6	10	4	9
*F*_15_	min	2.55 × 10^4^	8.54 × 10^3^	1.41 × 10^9^	9.41 × 10^5^	5.32 × 10^5^	4.45 × 10^9^	4.27 × 10^8^	6.06 × 10^9^	2.59 × 10^8^	3.87 × 10^9^
	std	2.80 × 10^4^	6.41 × 10^4^	2.99 × 10^9^	2.15 × 10^7^	1.35 × 10^8^	3.91 × 10^9^	5.05 × 10^9^	4.27 × 10^9^	1.30 × 10^9^	1.13 × 10^10^
	avg	6.26 × 10^4^	3.98 × 10^4^	5.39 × 10^9^	1.50 × 10^7^	5.22 × 10^7^	1.13 × 10^10^	6.87 × 10^9^	1.61 × 10^10^	1.76 × 10^9^	1.87 × 10^10^
	rank	2	1	6	3	4	8	7	9	5	10
*F*_16_	min	2.93 × 10^3^	3.70 × 10^3^	5.63 × 10^3^	4.29 × 10^3^	4.59 × 10^3^	7.12 × 10^3^	5.75 × 10^3^	9.30 × 10^3^	5.24 × 10^3^	7.62 × 10^3^
	std	6.05 × 10^2^	4.82 × 10^2^	1.51 × 10^3^	9.73 × 10^2^	5.24 × 10^2^	1.57 × 10^3^	9.66 × 10^2^	2.38 × 10^3^	5.77 × 10^2^	3.18 × 10^3^
	avg	4.11 × 10^3^	4.59 × 10^3^	8.05 × 10^3^	6.23 × 10^3^	5.45 × 10^3^	9.69 × 10^3^	7.54 × 10^3^	1.33 × 10^4^	6.34 × 10^3^	1.20 × 10^4^
	rank	1	2	7	4	3	8	6	10	5	9
*F*_17_	min	2.85 × 10^3^	3.00 × 10^3^	4.29 × 10^3^	3.38 × 10^3^	3.40 × 10^3^	5.03 × 10^3^	4.03 × 10^3^	6.45 × 10^3^	4.13 × 10^3^	5.62 × 10^3^
	std	3.48 × 10^2^	3.86 × 10^2^	4.72 × 10^3^	5.14 × 10^2^	7.39 × 10^2^	7.20 × 10^3^	3.07 × 10^4^	4.01 × 10^4^	2.32 × 10^3^	4.74 × 10^5^
	avg	3.48 × 10^3^	3.73 × 10^3^	1.17 × 10^4^	4.28 × 10^3^	4.48 × 10^3^	1.20 × 10^4^	2.26 × 10^4^	5.70 × 10^4^	5.81 × 10^3^	2.78 × 10^5^
	rank	1	2	6	3	4	7	8	9	5	10
*F*_18_	min	4.07 × 10^5^	5.01 × 10^5^	3.18 × 10^7^	2.14 × 10^6^	2.90 × 10^6^	3.82 × 10^7^	6.50 × 10^6^	1.71 × 10^8^	1.04 × 10^7^	2.00 × 10^7^
	std	1.80 × 10^6^	3.48 × 10^6^	1.29 × 10^8^	3.34 × 10^7^	6.66 × 10^7^	8.96 × 10^7^	1.46 × 10^8^	2.88 × 10^8^	3.97 × 10^7^	3.70 × 10^8^
	avg	2.95 × 10^6^	3.84 × 10^6^	1.78 × 10^8^	4.61 × 10^7^	7.10 × 10^7^	1.86 × 10^8^	1.76 × 10^8^	5.97 × 10^8^	7.30 × 10^7^	6.23 × 10^8^
	rank	1	2	7	3	4	8	6	9	5	10
*F*_19_	min	8.13 × 10^4^	3.14 × 10^4^	1.04 × 10^9^	4.41 × 10^5^	3.36 × 10^6^	6.98 × 10^8^	8.04 × 10^7^	2.48 × 10^9^	1.97 × 10^8^	1.72 × 10^9^
	std	6.39 × 10^5^	7.02 × 10^5^	1.68 × 10^9^	6.72 × 10^6^	2.23 × 10^7^	1.41 × 10^9^	3.90 × 10^9^	2.54 × 10^9^	5.71 × 10^8^	3.63 × 10^9^
	avg	7.60 × 10^5^	6.66 × 10^5^	4.76 × 10^9^	8.33 × 10^6^	3.05 × 10^7^	3.74 × 10^9^	4.64 × 10^9^	6.99 × 10^9^	9.22 × 10^8^	7.65 × 10^9^
	rank	2	1	8	3	4	6	7	9	5	10
*F*_20_	min	2.48 × 10^3^	3.49 × 10^3^	2.93 × 10^3^	3.20 × 10^3^	3.26 × 10^3^	3.62 × 10^3^	3.51 × 10^3^	4.19 × 10^3^	3.52 × 10^3^	4.19 × 10^3^
	std	4.32 × 10^2^	1.31 × 10^2^	2.86 × 10^2^	3.00 × 10^2^	3.82 × 10^2^	1.96 × 10^2^	4.20 × 10^2^	2.50 × 10^2^	3.64 × 10^2^	3.49 × 10^2^
	avg	3.44 × 10^3^	3.76 × 10^3^	3.71 × 10^3^	3.91 × 10^3^	4.14 × 10^3^	4.16 × 10^3^	4.30 × 10^3^	4.90 × 10^3^	4.20 × 10^3^	5.29 × 10^3^
	rank	1	3	2	4	5	6	8	9	7	10
*F*_21_	min	2.54 × 10^3^	2.68 × 10^3^	2.94 × 10^3^	2.86 × 10^3^	3.19 × 10^3^	3.08 × 10^3^	2.97 × 10^3^	3.26 × 10^3^	2.88 × 10^3^	3.31 × 10^3^
	std	8.75 × 10^1^	1.14 × 10^2^	8.45 × 10^1^	1.30 × 10^2^	9.90 × 10^1^	8.68 × 10^1^	6.42 × 10^1^	9.39 × 10^1^	4.95 × 10^1^	1.17 × 10^2^
	avg	2.70 × 10^3^	2.92 × 10^3^	3.10 × 10^3^	3.04 × 10^3^	3.32 × 10^3^	3.20 × 10^3^	3.09 × 10^3^	3.47 × 10^3^	2.97 × 10^3^	3.50 × 10^3^
	rank	1	2	6	4	8	7	5	9	3	10
*F*_22_	min	2.33 × 10^3^	9.63 × 10^3^	1.36 × 10^4^	1.14 × 10^4^	1.51 × 10^4^	1.63 × 10^4^	1.53 × 10^4^	1.75 × 10^4^	1.47 × 10^4^	1.72 × 10^4^
	std	3.83 × 10^3^	2.15 × 10^3^	6.55 × 10^2^	1.18 × 10^3^	7.26 × 10^2^	4.33 × 10^2^	8.36 × 10^2^	5.65 × 10^2^	9.70 × 10^2^	4.99 × 10^2^
	avg	1.07 × 10^4^	1.26 × 10^4^	1.62 × 10^4^	1.42 × 10^4^	1.65 × 10^4^	1.69 × 10^4^	1.66 × 10^4^	1.88 × 10^4^	1.62 × 10^4^	1.84 × 10^4^
	rank	1	2	4	3	6	8	7	10	5	9
*F*_23_	min	3.13 × 10^3^	3.58 × 10^3^	4.05 × 10^3^	3.33 × 10^3^	3.75 × 10^3^	4.12 × 10^3^	3.91 × 10^3^	4.35 × 10^3^	3.58 × 10^3^	3.90 × 10^3^
	std	1.60 × 10^2^	2.56 × 10^2^	2.51 × 10^2^	1.67 × 10^2^	1.96 × 10^2^	2.04 × 10^2^	1.95 × 10^2^	2.22 × 10^2^	1.58 × 10^2^	3.59 × 10^2^
	avg	3.39 × 10^3^	4.11 × 10^3^	4.47 × 10^3^	3.79 × 10^3^	4.10 × 10^3^	4.50 × 10^3^	4.15 × 10^3^	4.85 × 10^3^	3.86 × 10^3^	4.40 × 10^3^
	rank	1	5	8	2	4	9	6	10	3	7
*F*_24_	min	3.32 × 10^3^	3.57 × 10^3^	4.10 × 10^3^	3.44 × 10^3^	4.14 × 10^3^	4.31 × 10^3^	3.87 × 10^3^	4.92 × 10^3^	3.84 × 10^3^	3.91 × 10^3^
	std	2.20 × 10^2^	4.75 × 10^2^	3.30 × 10^2^	1.54 × 10^2^	2.28 × 10^2^	2.83 × 10^2^	2.23 × 10^2^	3.41 × 10^2^	1.72 × 10^2^	2.43 × 10^2^
	avg	3.66 × 10^3^	4.56 × 10^3^	4.94 × 10^3^	3.79 × 10^3^	4.55 × 10^3^	4.88 × 10^3^	4.41 × 10^3^	5.77 × 10^3^	4.12 × 10^3^	4.28 × 10^3^
	rank	1	7	9	2	6	8	5	10	3	4
*F*_25_	min	3.08 × 10^3^	3.16 × 10^3^	1.34 × 10^4^	3.63 × 10^3^	6.74 × 10^3^	1.19 × 10^4^	1.22 × 10^4^	1.57 × 10^4^	9.05 × 10^3^	1.81 × 10^4^
	std	4.69 × 10^1^	4.31 × 10^1^	1.09 × 10^3^	2.36 × 10^2^	4.83 × 10^3^	1.44 × 10^3^	1.85 × 10^3^	1.02 × 10^3^	1.48 × 10^3^	1.70 × 10^4^
	avg	3.19 × 10^3^	3.24 × 10^3^	1.55 × 10^4^	4.11 × 10^3^	1.25 × 10^4^	1.54 × 10^4^	1.62 × 10^4^	1.78 × 10^4^	1.18 × 10^4^	4.24 × 10^4^
	rank	1	2	7	3	5	6	8	9	4	10
*F*_26_	min	3.52 × 10^3^	4.48 × 10^3^	1.38 × 10^4^	5.43 × 10^3^	1.31 × 10^4^	1.62 × 10^4^	1.53 × 10^4^	1.79 × 10^4^	1.43 × 10^4^	1.48 × 10^4^
	std	2.97 × 10^3^	2.73 × 10^3^	1.19 × 10^3^	2.18 × 10^3^	1.25 × 10^3^	5.95 × 10^2^	1.18 × 10^3^	4.16 × 10^2^	7.81 × 10^2^	4.13 × 10^3^
	avg	8.50 × 10^3^	9.79 × 10^3^	1.65 × 10^4^	1.38 × 10^4^	1.50 × 10^4^	1.75 × 10^4^	1.80 × 10^4^	1.91 × 10^4^	1.56 × 10^4^	2.37 × 10^4^
	rank	1	2	6	3	4	7	8	9	5	10
*F*_27_	min	3.52 × 10^3^	4.13 × 10^3^	5.22 × 10^3^	4.05 × 10^3^	4.56 × 10^3^	5.14 × 10^3^	4.88 × 10^3^	7.51 × 10^3^	4.38 × 10^3^	4.90 × 10^3^
	std	1.57 × 10^2^	8.34 × 10^2^	8.13 × 10^2^	6.40 × 10^2^	4.25 × 10^2^	7.86 × 10^2^	5.43 × 10^2^	1.12 × 10^3^	4.49 × 10^2^	8.11 × 10^2^
	avg	3.76 × 10^3^	5.30 × 10^3^	6.89 × 10^3^	4.89 × 10^3^	5.49 × 10^3^	6.78 × 10^3^	5.83 × 10^3^	9.40 × 10^3^	5.14 × 10^3^	6.13 × 10^3^
	rank	1	4	9	2	5	8	6	10	3	7
*F*_28_	min	3.43 × 10^3^	3.59 × 10^3^	1.00 × 10^4^	4.09 × 10^3^	6.67 × 10^3^	1.18 × 10^4^	8.78 × 10^3^	1.26 × 10^4^	8.80 × 10^3^	1.22 × 10^4^
	std	6.77 × 10^1^	1.86 × 10^2^	1.26 × 10^3^	4.95 × 10^2^	1.88 × 10^3^	1.66 × 10^3^	1.89 × 10^3^	1.91 × 10^3^	9.44 × 10^2^	3.01 × 10^3^
	avg	3.62 × 10^3^	3.88 × 10^3^	1.23 × 10^4^	5.10 × 10^3^	9.94 × 10^3^	1.45 × 10^4^	1.25 × 10^4^	1.67 × 10^4^	1.02 × 10^4^	1.95 × 10^4^
	rank	1	2	6	3	4	8	7	9	5	10
*F*_29_	min	4.33 × 10^3^	5.57 × 10^3^	9.93 × 10^3^	6.79 × 10^3^	5.71 × 10^3^	1.14 × 10^4^	9.33 × 10^3^	2.06 × 10^4^	7.97 × 10^3^	9.74 × 10^3^
	std	7.06 × 10^2^	7.96 × 10^2^	4.36 × 10^4^	1.71 × 10^3^	8.22 × 10^2^	2.17 × 10^5^	7.95 × 10^4^	1.01 × 10^6^	9.07 × 10^3^	8.32 × 10^5^
	avg	5.57 × 10^3^	6.59 × 10^3^	5.48 × 10^4^	9.19 × 10^3^	7.17 × 10^3^	1.61 × 10^5^	5.85 × 10^4^	8.73 × 10^5^	1.63 × 10^4^	4.12 × 10^5^
	rank	1	2	6	4	3	8	7	10	5	9
*F*_30_	min	2.15 × 10^7^	4.10 × 10^7^	9.81 × 10^8^	8.38 × 10^7^	1.64 × 10^8^	1.15 × 10^9^	5.61 × 10^8^	5.19 × 10^9^	4.66 × 10^8^	4.32 × 10^9^
	std	1.03 × 10^7^	2.76 × 10^7^	3.21 × 10^9^	1.02 × 10^8^	1.41 × 10^8^	3.62 × 10^9^	4.54 × 10^9^	4.11 × 10^9^	7.83 × 10^8^	5.23 × 10^9^
	avg	4.04 × 10^7^	8.13 × 10^7^	6.39 × 10^9^	2.27 × 10^8^	3.59 × 10^8^	7.89 × 10^9^	6.25 × 10^9^	1.33 × 10^10^	1.84 × 10^9^	1.31 × 10^10^
	rank	1	2	7	3	4	8	6	9	5	10

**Table 3:** Wilcoxon results for CEC2017.

*p*-Value Interpretation	CPO	AOA	WOA	GA	COA	MShOA	LCA	CFOA	SFOA
>0.05	3	9	9	9	0	0	0	9	0
<0.05	26	20	20	20	29	29	29	20	29

**Table 4:** CEC2022 test function results.

Function	Type	ACDCPO	CPO	AOA	WOA	GA	COA	MShOA	LCA	CFOA	SFOA
*F*_1_	min	1.17 × 10^3^	1.38 × 10^4^	1.90 × 10^4^	1.40 × 10^4^	4.40 × 10^4^	2.15 × 10^4^	2.01 × 10^4^	3.31 × 10^4^	2.26 × 10^4^	4.03 × 10^4^
	std	2.68 × 10^3^	2.26 × 10^4^	9.62 × 10^3^	1.57 × 10^4^	2.51 × 10^4^	1.06 × 10^4^	7.36 × 10^3^	3.52 × 10^4^	1.46 × 10^4^	1.39 × 10^5^
	avg	4.32 × 10^3^	4.50 × 10^4^	3.49 × 10^4^	2.86 × 10^4^	9.08 × 10^4^	4.07 × 10^4^	3.32 × 10^4^	1.04 × 10^5^	4.40 × 10^4^	1.66 × 10^5^
	rank	1	7	4	2	8	5	3	9	6	10
*F*_2_	min	4.46 × 10^2^	4.49 × 10^2^	1.03 × 10^3^	4.83 × 10^2^	4.89 × 10^2^	1.50 × 10^3^	1.00 × 10^3^	1.23 × 10^3^	8.29 × 10^2^	1.77 × 10^3^
	std	1.47 × 10^1^	1.64 × 10^1^	9.77 × 10^2^	5.95 × 10^1^	5.94 × 10^1^	6.62 × 10^2^	1.15 × 10^3^	1.42 × 10^3^	2.33 × 10^2^	3.19 × 10^3^
	avg	4.62 × 10^2^	4.68 × 10^2^	2.38 × 10^3^	5.70 × 10^2^	5.74 × 10^2^	2.85 × 10^3^	2.35 × 10^3^	4.24 × 10^3^	1.20 × 10^3^	4.90 × 10^3^
	rank	1	2	7	3	4	8	6	9	5	10
*F*_3_	min	6.08 × 10^2^	6.42 × 10^2^	6.46 × 10^2^	6.49 × 10^2^	6.79 × 10^2^	6.50 × 10^2^	6.39 × 10^2^	6.75 × 10^2^	6.40 × 10^2^	6.42 × 10^2^
	std	1.70 × 10^1^	9.22 × 10^0^	1.01 × 10^1^	1.01 × 10^1^	1.63 × 10^1^	1.03 × 10^1^	1.44 × 10^1^	1.04 × 10^1^	1.04 × 10^1^	2.80 × 10^1^
	avg	6.31 × 10^2^	6.67 × 10^2^	6.64 × 10^2^	6.66 × 10^2^	7.06 × 10^2^	6.75 × 10^2^	6.72 × 10^2^	7.00 × 10^2^	6.60 × 10^2^	7.10 × 10^2^
	rank	1	5	3	4	9	7	6	8	2	10
*F*_4_	min	8.30 × 10^2^	8.83 × 10^2^	9.16 × 10^2^	8.79 × 10^2^	9.88 × 10^2^	9.42 × 10^2^	9.16 × 10^2^	9.81 × 10^2^	8.89 × 10^2^	9.66 × 10^2^
	std	2.99 × 10^1^	4.48 × 10^0^	1.71 × 10^1^	2.71 × 10^1^	2.37 × 10^1^	1.35 × 10^1^	2.28 × 10^1^	2.01 × 10^1^	2.06 × 10^1^	3.97 × 10^1^
	avg	8.76 × 10^2^	8.89 × 10^2^	9.50 × 10^2^	9.21 × 10^2^	1.03 × 10^3^	9.68 × 10^2^	9.63 × 10^2^	1.02 × 10^3^	9.42 × 10^2^	1.08 × 10^3^
	rank	1	2	5	3	9	7	6	8	4	10
*F*_5_	min	1.17 × 10^3^	1.38 × 10^4^	1.90 × 10^4^	1.40 × 10^4^	4.40 × 10^4^	2.15 × 10^4^	2.01 × 10^4^	3.31 × 10^4^	2.26 × 10^4^	4.03 × 10^4^
	std	2.68 × 10^3^	2.26 × 10^4^	9.62 × 10^3^	1.57 × 10^4^	2.51 × 10^4^	1.06 × 10^4^	7.36 × 10^3^	3.52 × 10^4^	1.46 × 10^4^	1.39 × 10^5^
	avg	4.32 × 10^3^	4.50 × 10^4^	3.49 × 10^4^	2.86 × 10^4^	9.08 × 10^4^	4.07 × 10^4^	3.32 × 10^4^	1.04 × 10^5^	4.40 × 10^4^	1.66 × 10^5^
	rank	2	5	3	8	1	6	7	9	4	10
*F*_6_	min	2.16 × 10^3^	1.97 × 10^3^	2.29 × 10^5^	2.30 × 10^4^	2.31 × 10^3^	4.28 × 10^8^	6.47 × 10^7^	1.31 × 10^9^	1.22 × 10^7^	4.11 × 10^8^
	std	2.08 × 10^3^	1.72 × 10^3^	7.60 × 10^8^	1.90 × 10^6^	3.08 × 10^4^	1.26 × 10^9^	1.39 × 10^9^	1.37 × 10^9^	3.42 × 10^8^	2.89 × 10^9^
	avg	4.78 × 10^3^	3.80 × 10^3^	8.06 × 10^8^	1.19 × 10^6^	2.04 × 10^4^	2.43 × 10^9^	1.26 × 10^9^	4.64 × 10^9^	2.79 × 10^8^	4.49 × 10^9^
	rank	2	1	6	4	3	8	7	10	5	9
*F*_7_	min	2.04 × 10^3^	2.10 × 10^3^	2.15 × 10^3^	2.11 × 10^3^	2.15 × 10^3^	2.15 × 10^3^	2.08 × 10^3^	2.17 × 10^3^	2.12 × 10^3^	2.24 × 10^3^
	std	4.57 × 10^1^	1.55 × 10^2^	6.89 × 10^1^	5.10 × 10^1^	7.82 × 10^1^	4.11 × 10^1^	8.52 × 10^1^	1.09 × 10^2^	4.22 × 10^1^	9.24 × 10^1^
	avg	2.13 × 10^3^	2.31 × 10^3^	2.22 × 10^3^	2.20 × 10^3^	2.27 × 10^3^	2.21 × 10^3^	2.20 × 10^3^	2.42 × 10^3^	2.17 × 10^3^	2.41 × 10^3^
	rank	1	8	6	3	7	5	4	10	2	9
*F*_8_	min	2.22 × 10^3^	2.22 × 10^3^	2.26 × 10^3^	2.24 × 10^3^	2.24 × 10^3^	2.24 × 10^3^	2.24 × 10^3^	2.43 × 10^3^	2.25 × 10^3^	2.41 × 10^3^
	std	5.82 × 10^1^	2.02 × 10^2^	2.45 × 10^2^	9.76 × 10^1^	6.97 × 10^1^	1.01 × 10^2^	8.53 × 10^1^	6.71 × 10^2^	1.33 × 10^2^	4.56 × 10^3^
	avg	2.28 × 10^3^	2.51 × 10^3^	2.54 × 10^3^	2.31 × 10^3^	2.33 × 10^3^	2.33 × 10^3^	2.31 × 10^3^	3.23 × 10^3^	2.44 × 10^3^	4.50 × 10^3^
	rank	1	7	8	2	4	5	3	9	6	10
*F*_9_	min	1.17 × 10^3^	1.38 × 10^4^	1.90 × 10^4^	1.40 × 10^4^	4.40 × 10^4^	2.15 × 10^4^	2.01 × 10^4^	3.31 × 10^4^	2.26 × 10^4^	4.03 × 10^4^
	std	2.68 × 10^3^	2.26 × 10^4^	9.62 × 10^3^	1.57 × 10^4^	2.51 × 10^4^	1.06 × 10^4^	7.36 × 10^3^	3.52 × 10^4^	1.46 × 10^4^	1.39 × 10^5^
	avg	4.32 × 10^3^	4.50 × 10^4^	3.49 × 10^4^	2.86 × 10^4^	9.08 × 10^4^	4.07 × 10^4^	3.32 × 10^4^	1.04 × 10^5^	4.40 × 10^4^	1.66 × 10^5^
	rank	1	2	7	3	4	8	6	10	5	9
*F*_10_	min	2.50 × 10^3^	2.50 × 10^3^	3.49 × 10^3^	2.50 × 10^3^	2.57 × 10^3^	2.63 × 10^3^	2.54 × 10^3^	7.05 × 10^3^	2.55 × 10^3^	5.30 × 10^3^
	std	1.31 × 10^3^	1.51 × 10^3^	7.25 × 10^2^	1.10 × 10^3^	1.02 × 10^3^	1.06 × 10^3^	1.60 × 10^3^	3.90 × 10^2^	1.87 × 10^3^	5.68 × 10^2^
	avg	4.40 × 10^3^	5.70 × 10^3^	5.77 × 10^3^	4.78 × 10^3^	6.06 × 10^3^	6.35 × 10^3^	4.31 × 10^3^	7.81 × 10^3^	4.65 × 10^3^	7.49 × 10^3^
	rank	2	5	6	4	7	8	1	10	3	9
*F*_11_	min	2.61 × 10^3^	2.60 × 10^3^	6.19 × 10^3^	3.11 × 10^3^	5.05 × 10^3^	7.45 × 10^3^	7.71 × 10^3^	8.22 × 10^3^	1.28 × 10^4^	4.22 × 10^4^
	std	8.63 × 10^1^	1.82 × 10^2^	2.40 × 10^3^	2.48 × 10^2^	1.58 × 10^3^	5.74 × 10^2^	7.38 × 10^2^	4.73 × 10^2^	4.85 × 10^3^	6.47 × 10^4^
	avg	2.94 × 10^3^	3.05 × 10^3^	8.70 × 10^3^	3.49 × 10^3^	6.86 × 10^3^	8.92 × 10^3^	9.12 × 10^3^	1.02 × 10^4^	2.01 × 10^4^	1.64 × 10^5^
	rank	1	2	5	3	4	6	7	8	9	10
*F*_12_	min	2.94 × 10^3^	3.03 × 10^3^	3.45 × 10^3^	2.96 × 10^3^	3.21 × 10^3^	3.12 × 10^3^	3.18 × 10^3^	3.69 × 10^3^	3.04 × 10^3^	3.02 × 10^3^
	std	4.63 × 10^1^	2.96 × 10^2^	2.00 × 10^2^	1.07 × 10^2^	1.33 × 10^2^	2.23 × 10^2^	1.95 × 10^2^	3.44 × 10^2^	9.27 × 10^1^	1.49 × 10^2^
	avg	3.00 × 10^3^	3.32 × 10^3^	3.83 × 10^3^	3.07 × 10^3^	3.50 × 10^3^	3.62 × 10^3^	3.41 × 10^3^	4.38 × 10^3^	3.18 × 10^3^	3.33 × 10^3^
	rank	1	4	9	2	7	8	6	10	3	5

**Table 5:** Wilcoxon results for CEC2022.

*p*-Value Interpretation	CPO	AOA	WOA	GA	COA	MShOA	LCA	CFOA	SFOA
>0.05	2	0	1	0	0	1	0	2	0
<0.05	10	12	11	12	12	11	12	10	12

**Table 6:** Statistical results of cantilever beam design problem experiment.

Algorithm	Best	Worst	Std	Mean	Median
ACDCPO	1.3404	1.3606	0.0075	1.3504	1.3502
CPO	1.391	1.6428	0.0765	1.4821	1.4698
AOA	2.2166	6.8747	1.511	3.4719	2.8548
WOA	1.4774	3.2486	0.5254	1.9334	1.8017
GA	1.366	1.4815	0.0336	1.4166	1.4039
COA	1.3659	1.5341	0.0531	1.4541	1.4609
MShOA	1.4234	1.7282	0.0966	1.5662	1.5862
LCA	1.4718	1.6228	0.0477	1.5754	1.5791
CFOA	4.2103	7.9981	1.0883	6.4668	6.5807
SFOA	4.0339	10.2091	2.2433	7.6749	8.2114

**Table 7:** Statistical results of pressure vessel design problem experiment.

Algorithm	Best	Worst	Std	Mean	Median
ACDCPO	6605.754354	7820.946041	454.8553237	7160.757748	7104.061166
CPO	6865.62155	35,079.51869	9499.130234	17,454.92254	14,685.46213
AOA	7166.862905	14,472.166	2217.436	9887.637892	9632.555788
WOA	7789.106457	19,831.66438	4571.594315	11,932.2791	9707.2581
GA	6590.808231	8972.166419	655.7714015	7956.185669	7858.877646
COA	6543.031889	10,842.75189	1284.336968	8573.012939	8707.261273
MShOA	6711.495404	8753.407616	701.7130386	7467.194957	7445.343063
LCA	11,026.31946	19,328.11873	2412.722028	16,108.82781	16,900.63019
CFOA	6474.510625	22,390.29452	5562.111548	11,998.21729	10,094.90118
SFOA	9886.468785	65,313.84564	16,921.5095	32,238.5248	31,245.23711

**Table 8:** Statistical results of stepped cone pulley problem experiment.

Algorithm	Best	Worst	Std	Mean	Median
ACDCPO	6443.29855	677,038.1808	217,016.9128	262,749.5508	241,819.1716
CPO	49,473.33984	7.5978 × 10^11^	3.2635 × 10^11^	5.24811 × 10^11^	7.59741 × 10^11^
AOA	3,276,041,666	1.43758 × 10^11^	49,359,995,568	62,434,798,920	51,919,286,815
WOA	6159.882531	2.80394 × 10^11^	88,119,916,309	31,259,231,406	27,408.44632
GA	9432.002819	4,044,673,229	1,376,220,494	742,081,675.1	75,979,461.39
COA	75,608,744,090	6.92283 × 10^12^	2.03656 × 10^12^	1.20517 × 10^12^	7.8904 × 10^11^
MShOA	1.47527×10^12^	3.62634 × 10^13^	1.07518 × 10^13^	9.51688 × 10^12^	6.62283 × 10^12^
LCA	2.55999×10^18^	3.04094 × 10^20^	9.11373 × 10^19^	5.66972 × 10^19^	1.87751 × 10^19^
CFOA	2,854,608,155	7.41441 × 10^17^	2.3478 × 10^17^	9.14773 × 10^16^	8.73621 × 10^11^
SFOA	8.42264×10^11^	7.19319 × 10^12^	2.51282 × 10^12^	2.39512 × 10^12^	1.12943 × 10^12^

**Table 9:** Statistical results of the industrial refrigeration system optimization design problem.

Algorithm	Best	Worst	Std	Mean	Median
ACDCPO	62.19929413	1.03456 × 10^15^	3.27157 × 10^14^	1.03456 × 10^14^	111,128.8028
CPO	11.836867	4.62926 × 10^16^	2.00606 × 10^16^	1.91764 × 10^16^	1.72538 × 10^16^
AOA	7.759928387	1.82783 × 10^15^	6.03606 × 10^14^	3.86323 × 10^14^	3700.160337
WOA	0.145987551	2.78107 × 10^16^	1.10681 × 10^16^	6.77902 × 10^15^	1.05412 × 10^15^
GA	0.074432405	1.09577 × 10^16^	3.46514 × 10^15^	1.09577 × 10^15^	0.250723389
COA	1.40779×10^14^	5.1585 × 10^16^	1.9295 × 10^16^	2.21806 × 10^16^	1.68064 × 10^16^
MShOA	4.9677×10^14^	1.12483 × 10^16^	3.76324 × 10^15^	6.00583 × 10^15^	6.90405 × 10^15^
LCA	1.89882×10^16^	1.68817 × 10^18^	5.34667 × 10^17^	4.72954 × 10^17^	2.75491 × 10^17^
CFOA	8.03136×10^15^	4.46209 × 10^16^	1.37702 × 10^16^	1.85314 × 10^16^	1.21543 × 10^16^
SFOA	1.36469×10^15^	5.71333 × 10^16^	1.57557 × 10^16^	3.94669 × 10^16^	4.19247 × 10^16^

**Table 10:** Comparison results of evaluation indicators.

Model	MAE	MAPE	MSE	RMSE	R^2^
BP	33.681	9.3395	2011.0	44.012	86.926
CPO-BP	31.183	8.712	1540.1	38.816	89.126
GA-BP	30.024	8.3163	1425.5	37.576	89.936
AOA-BP	29.975	8.226	1405.9	37.233	90.074
COA-BP	32.528	9.0969	1714.3	40.677	87.896
LCA-BP	32.080	9.0222	1621.2	39.960	88.553
CFOA-BP	31.266	8.6928	1555.9	39.191	89.014
WOA-BP	30.804	8.5183	1531.5	38.647	89.187
SFOA-BP	29.348	8.1881	1352.1	36.810	90.453
MShOA-BP	29.867	8.295	1371.6	36.886	90.315
ACDCPO-BP	28.735	8.0408	1351.6	36.781	91.211

## Data Availability

The original contributions presented in this study are included in the article. Further inquiries can be directed to the corresponding author.

## Abbreviations

The following abbreviations are used in this manuscript:

CPO	Chinese Pangolin Optimizer
BP	Back-propagation
GA	Genetic Algorithm
PSO	Particle Swarm Optimization
ABC	Artificial Bee Colony
BA	Bat Algorithm
GWO	Grey Wolf Optimizer
ZOA	Zebra Optimization Algorithm
GNS	Good Nodes Set
WOA	Whale Optimization Algorithm
AOA	Arithmetic Optimization Algorithm
COA	Cuckoo Optimization Algorithm
CFOA	Fishing Optimizer Algorithm
LCA	Liver Cancer Optimization Algorithm
MShOA	Mantis Shrimp Optimization Algorithm
SFOA	Superb Fairy-wren Optimization Algorithm
CEC	Congress on Evolutionary Computation
MAE	Mean Absolute Error
MAPE	Mean Absolute Percentage Error
MSE	Mean Squared Error
RMSE	Root Mean Squared Error
